# A Multiparametric Method Based on Clinical and CT-Based Radiomics to Predict the Expression of p53 and VEGF in Patients With Spinal Giant Cell Tumor of Bone

**DOI:** 10.3389/fonc.2022.894696

**Published:** 2022-06-21

**Authors:** Qizheng Wang, Yang Zhang, Enlong Zhang, Xiaoying Xing, Yongye Chen, Ke Nie, Huishu Yuan, Min-Ying Su, Ning Lang

**Affiliations:** ^1^ Department of Radiology, Peking University Third Hospital, Beijing, China; ^2^ Department of Radiological Sciences, University of California Irvine, Irvine, CA, United States; ^3^ Department of Radiation Oncology, Robert Wood Johnson Medical School, New Brunswick, NJ, United States; ^4^ Department of Radiology, Peking University International Hospital, Beijing, China; ^5^ Department of Medical Imaging and Radiological Sciences, Kaohsiung Medical University, Kaohsiung, Taiwan

**Keywords:** tomography, quantitative imaging, giant cell tumor of bone, immunohistochemistry, tumor suppressor protein p53, vascular endothelial growth factors

## Abstract

**Purpose:**

This project aimed to assess the significance of vascular endothelial growth factor (VEGF) and p53 for predicting progression-free survival (PFS) in patients with spinal giant cell tumor of bone (GCTB) and to construct models for predicting these two biomarkers based on clinical and computer tomography (CT) radiomics to identify high-risk patients for improving treatment.

**Material and Methods:**

A retrospective study was performed from April 2009 to January 2019. A total of 80 patients with spinal GCTB who underwent surgery in our institution were identified. VEGF and p53 expression and clinical and general imaging information were collected. Multivariate Cox regression models were used to verify the prognostic factors. The radiomics features were extracted from the regions of interest (ROIs) in preoperative CT, and then important features were selected by the SVM to build classification models, evaluated by 10-fold crossvalidation. The clinical variables were processed using the same method to build a conventional model for comparison.

**Results:**

The immunohistochemistry of 80 patients was obtained: 49 with high-VEGF and 31 with low-VEGF, 68 with wild-type p53, and 12 with mutant p53. p53 and VEGF were independent prognostic factors affecting PFS found in multivariate Cox regression analysis. For VEGF, the Spinal Instability Neoplastic Score (SINS) was greater in the high than low groups, *p* < 0.001. For p53, SINS (*p* = 0.030) and Enneking stage (*p* = 0.017) were higher in mutant than wild-type groups. The VEGF radiomics model built using 3 features achieved an area under the curve (AUC) of 0.88, and the p53 radiomics model built using 4 features had an AUC of 0.79. The conventional model built using SINS, and the Enneking stage had a slightly lower AUC of 0.81 for VEGF and 0.72 for p53.

**Conclusion:**

p53 and VEGF are associated with prognosis in patients with spinal GCTB, and the radiomics analysis based on preoperative CT provides a feasible method for the evaluation of these two biomarkers, which may aid in choosing better management strategies.

## Introduction

Giant cell tumor of bone (GCTB) is one of the most common intermediate bone tumors, which occurs in young adults 20–40 years old with a high recurrence rate (20%–50%) (1) and a potential for aggressive behavior (2). Even if patients undergo the same surgical procedure and remove the tumor as completely as possible, the postoperative recurrence rate varies substantially. Many studies have suggested that this may be related to the aggressiveness of the tumor that each patient has, and thus, personalized stratified management is very important (3). For GCTB in the spine, postoperative recurrence is more common compared to GCTB in other bones, and it is also associated with a higher risk of malignant transformation (4, 5). During the surgery, it is necessary to protect the spinal cord and peripheral nerve function to minimize the postoperative complications caused by the resection damage; therefore, the tumor may not be completely resected to remain in a good quality of life (6). Given all these, it is important to characterize the aggressiveness of the spinal GCTB to choose an optimized personalized treatment. The genomics analysis and imaging may provide valuable information for treatment planning, postoperative monitoring, and prognosis assessment.

In 2020, the WHO updated the classification for primary musculoskeletal tumors, which reflects the knowledge generated from extensive research in the identification of novel gene alterations in many bone neoplasms (7). The change further emphasized that the assessment of bone tumors should be more thorough and personalized. We reviewed previous studies on prognostic-related molecular markers and found that the vascular endothelial growth factor (VEGF) and p53 mutation were two important biomarkers related to the evaluation of the biological aggressiveness of osteosarcoma and GCTB (3, 8–15). Angiogenesis occurs in numerous biological processes, which is essential for the growth of tumors and metastases. VEGF is one of the most important growth factors for the regulation of vascular development and angiogenesis (16, 17), which plays an important role in osteogenesis, bone repair, tumor cell development, and metastasis by stimulating angiogenesis (18). p53 is an important tumor suppressor gene in many carcinomas, and there are also extensive research studies for bone tumors (19). Mutation in p53 will lose this function and lead to tumorigenesis, which can also promote angiogenesis by regulating the expression of VEGF (20). For GCTB, high expression of VEGF (21–23) and mutant p53 (24–26) have been shown as risk factors for local recurrence and malignant transformation. However, the long-term follow-up studies focusing on these two biomarkers in spinal GCTB after total en bloc spondylectomy (TES), the current mainstream surgical method, were rarely reported.

The current assessment of preoperative spinal GCTB relies mainly on pathological and immunohistochemical examination of tissues taken by puncture biopsy. However, it is known that the analysis is not reliable in some cases because only a small amount of tissue in a large tumor is obtained. While this is sufficient for making a diagnosis, further characterization of molecular biomarkers may be limited by tumor heterogeneity. In addition, an invasive needle biopsy may lead to complications such as bleeding, fractures, and tumor metastasis. At present, surgeons also use some clinical scoring systems for preoperative assessment, such as the Spinal Instability Neoplastic Score (SINS) (27), the Visual Analog Scale (VAS) (28), and the Enneking stage (29). However, there is no research reporting how these scoring systems are related to the tumor biomarker status.

In recent years, “radiomics” has emerged as a widely used method to characterize diseases for molecular diagnosis, prognosis, and treatment monitoring by analyzing the spatial and temporal heterogeneity of tumors from medical images (30–35). As the full spatial extent of the tumor was considered, the computational techniques may provide a complimentary assessment of the whole tumor, thus overcoming the limitations of tissue sampling (36–38). Computed tomography (CT) is a cost-effective imaging method commonly used in the clinical examination of spinal tumors. The CT-based radiomics features may provide a new approach to reflect the heterogeneity of tumors related to the VEGF and p53 expression, which may make up for the limitations of preoperative puncture and provide supplemental information.

There are two main objectives in this study. The first aim is to evaluate the prognostic difference according to the status of VEGF and P53 using the progression-free survival (PFS) in a cohort of spinal GCTB patients with long-term follow-up. The second aim is then to build models based on preoperative CT to differentiate high vs. low VEGF and wild-type vs. mutant p53 to assist in preoperative tumor evaluation. Other information such as the clinically applied scoring system and traditional imaging evaluation results is included in the analysis, and the performance of the developed models is compared.

## Materials and Methods

### Patients

The study was approved by the Medical Science Research Ethics Committee, and the written informed consent was waived. We identified 105 consecutive patients with spinal GCTB at the orthopedics department between April 1, 2009, and January 1, 2019. The inclusion criteria were as follows (1) patients who had pathologically confirmed spinal GCTB; (2) preoperative CT was performed; and (3) the qualified postoperative pathological specimens were stored in the tissue bank.

**Figure 1:**
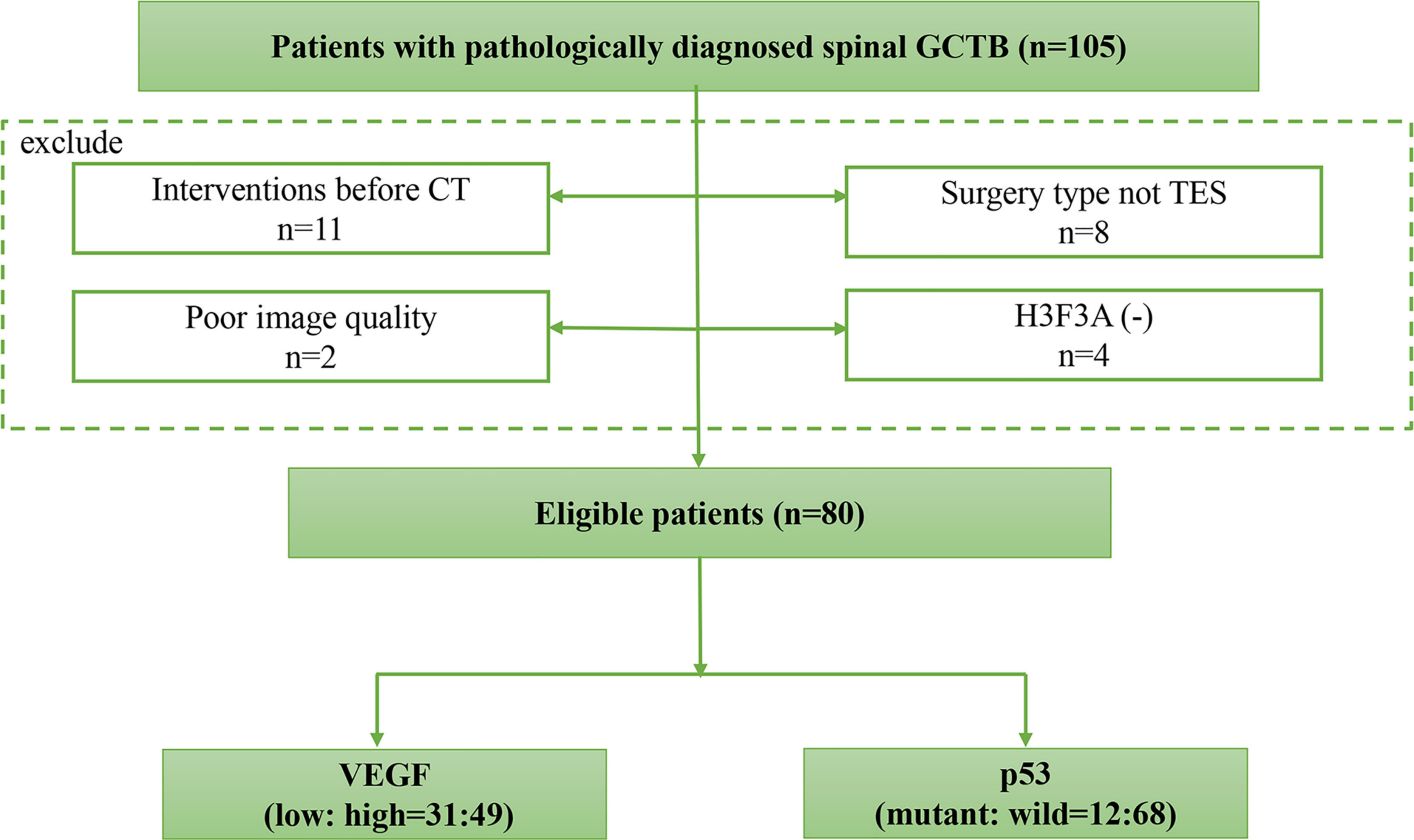
The subject identification flowchart. A total of 80 spinal GCTB cases with VEGF and p53 immunohistochemical staining results are included.

The exclusion criteria were as follows: (1) radiotherapy, preoperative neoadjuvant chemotherapy, or other interventions for lesions were performed before CT or surgery; (2) poor image quality due to susceptibility artifacts decided by radiologists; (3) the operation method was not TES; and (4) the immunohistochemical evaluation result of H3F3A was negative (37). Finally, a total of 80 patients were included, and their clinical and CT imaging data were collected. The subject identification flowchart is shown in **Figure 1**.

### Clinical and Imaging Characteristics

The clinical information for the preoperative evaluation of spinal tumors was obtained through the medical record system, including the symptom duration before surgery (months), SINS, VAS, and the Enneking stage. The instability was further defined based on the SINS into two categories: scores of 7 to 12 as indeterminate (possibly impending) instability and 13 to 18 as instability (39). The scoring methods are explained and shown in **Supplementary Part 1**.

### CT Imaging

CT imaging was performed using a GE Lightspeed 64-slice spiral CT (GE Medical System, Chalfont St Giles, UK) or a Siemens Somatom Definition Flash dual-source CT (Siemens, Erlangen, Germany). The parameters were 120 kVp, 200–300 mAs; collimator width of 0.625 or 0.60 mm; pitch of 1.0; slice thickness of 2 mm; and interlayer distance of 3 mm.

**Figure 2:**
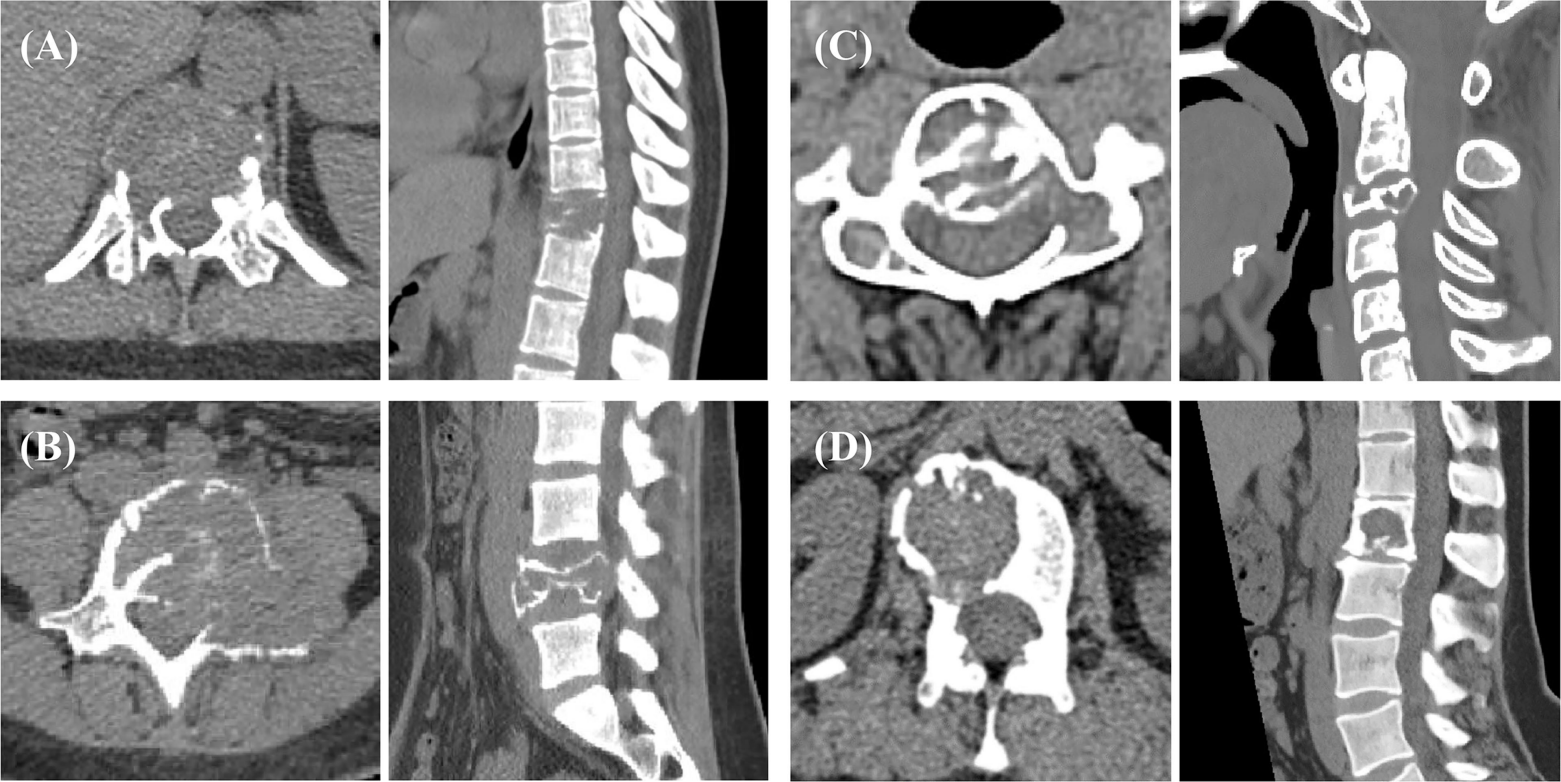
Spinal GCTB case examples from 4 patients. The boundary of the lesion can be clearly observed on the transverse images, which are used for tumor ROI drawing. The sagittal images show vertebral compression, spinal canal compression, and spinal stability, which are used to determine additional imaging features. The biomarker results of these patients: **(A)** mutant p53 and high VEGF; **(B)** mutant p53 and low VEGF; **(C)** wild-type p53 and high VEGF; and **(D)** wild-type p53 and low VEGF. The SINS of these 4 patients were 18, 11, 11, and 7.

For each case, 8 imaging features were determined: lesion location, position, vertebral compression, boundary, residual bone crest, “soap bubble sign”, largest diameter, and CT value. These were evaluated by 3 musculoskeletal radiologists, and the consensus results were used. The location of the lesion included the cervical, thoracic, lumbar, and sacral spine. The position was classified according to whether the lesion was located in the vertebral body or vertebral arch. The boundary was classified as clear or unclear. The “soap bubble sign” was defined as the bone cortex having obvious expansive changes compared with the normal vertebra. **Figure 2** shows the axial and sagittal images from 4 cases to illustrate the evaluation of imaging features.

### Evaluation of VEGF and p53 Expression

**Figure 3:**
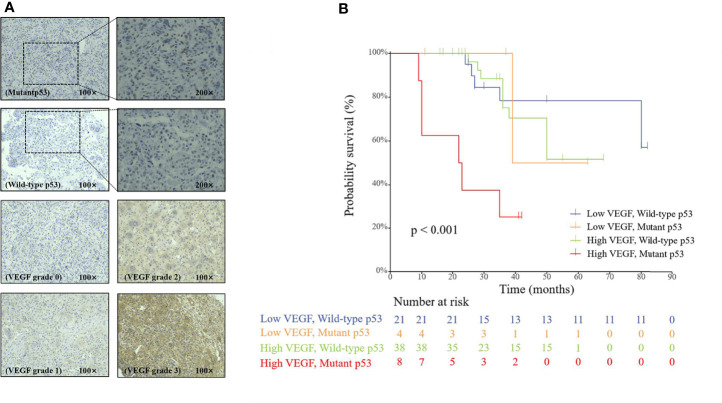
**(A)** Immunohistochemical staining of p53 and VEGF in spinal GCTB. **(B)** The Cox proportional hazards regression analysis of the p53 and VEGF groups.

The paraffin-embedded tissue block of the patient’s postoperative specimen was requested from the pathology department, and immunohistochemical staining of VEGF and p53 was performed by following the protocol (40). The expression levels of VEGF and p53 were independently evaluated by two experienced pathologists using a scoring system. The expression level of VEGF was divided into four grades according to the percentage of positively stained cells: ≤15% (grade 0), 15%–50% (grade 1), 50%–75% (grade 2), and ≥75% (grade 3). Since there were few cases of grades 0 and 3, grades 0–1 were classified into a low-VEGF group and grades 2–3 into a high-VEGF group. For p53, the tissue was considered positive when the proportion of nuclei positively stained for mutant p53 was >10%, otherwise negative. Examples of the immunohistochemical slides are illustrated in **Figure 3**.

### Tumor Segmentation on CT

For each case, the range of axial CT slices containing the tumor was first determined. The ROI of the tumor was manually delineated using the Image J software (National Institute of Health, Bethesda, USA) by a musculoskeletal radiologist (with 15 years of experience) and then validated by an experienced radiologist (with 25 years of experience in skeletal radiology). Discrepancies between the two radiologists were resolved by consensus. The two radiologists were not involved in the clinical and imaging characteristics evaluation and were blinded to other information about patients. The outlined ROIs on all imaging slices of a tumor were combined into a 3D tumor mask.

### Radiomics Analysis to Build Classification Model

**Figure 4:**
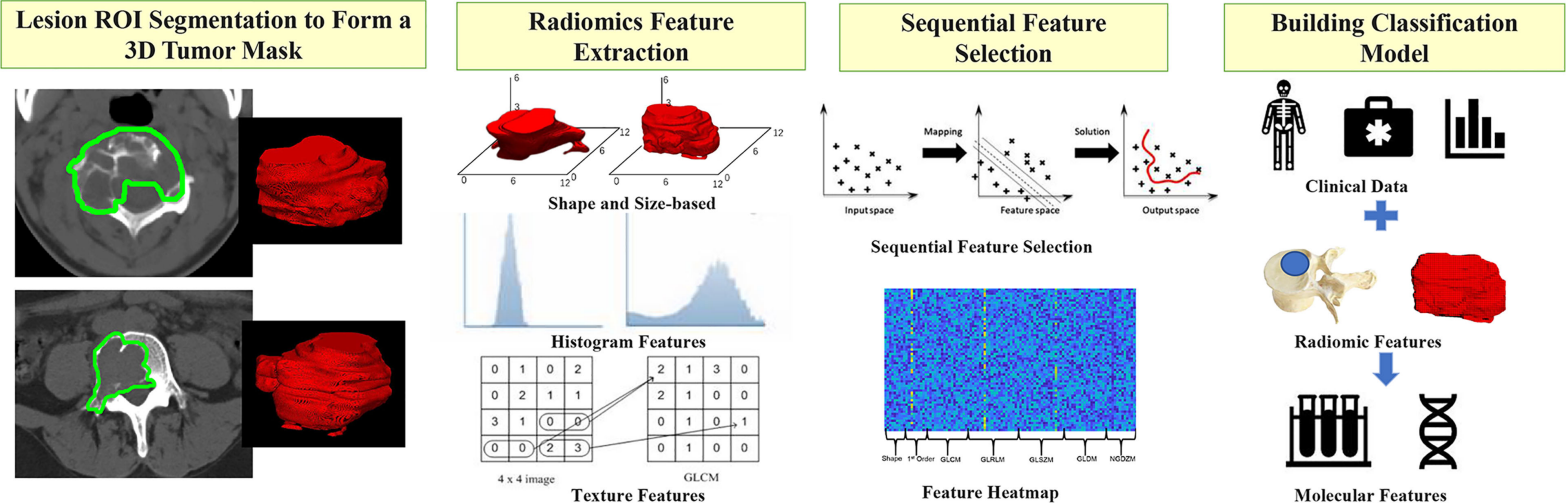
The radiomics analysis procedures to build the classification model. Step1: The lesion ROI is outlined on each slice and then combined into a 3D tumor mask. Step 2: PyRadiomics is applied to extract 107 features, including shape, first-order statistics, and texture from each tumor mask (GLCM is an example of a feature processing). Step 3: The sequential feature selection is performed by using SVM, and finally, Step 4: The SVM algorithm is applied to build the classification model.

The radiomics analysis procedures are illustrated in **Figure 4**. The feature extraction was done using PyRadiomics, an open-source Python package platform (http://www.radiomics.io/pyradiomics.html). For each patient, a total of 107 features, including shape, first-order statistics, and texture, were extracted. The list of features and how each feature is calculated is included in **Supplementary Material Part 2**. The segmented lesions on all 2D slices were rendered into a 3D space with isotropic voxel resolution for extracting the 3D texture features. Although using different quantization methods or wavelet transformation may generate many times features, they were highly correlated with the original features, so we only analyzed the original 107 features.

After the features were extracted, they were normalized to mean = 0 and standard deviation = 1. To evaluate the importance of these features in classification, the sequential feature selection process was done via the construction of multiple support vector machine (SVM) classifiers. In this process, we used SVM with a Gaussian kernel as the objective function to test the performance of models built with a subset of features. In the beginning, an empty candidate set was presented, and features were sequentially added. A 10-fold crossvalidation was applied to test the model performance. In each iteration, the training process was repeated 1,000 times to explore the robustness of each feature. After each iteration, the feature that led to the best performance was added to the candidate set. When the addition of features no longer met the criterion, the selection process stopped. Here, we used 10^−6^ as termination tolerance for the objective function value. The number of mutant p53 cases was much smaller than the wild-type p53, so we assigned different class weights according to the number of cases to address the issue of unbalanced classes. For the high vs. low VEGF, the case number was approximately equal.

The selected features were used to build the final SVM classification model with a Gaussian kernel to classify the high vs. low VEGF and wild-type vs. mutant p53 groups. The output of the model was a radiomics score (that is, a probability) for a case. The diagnostic performance was tested using 10-fold crossvalidation. Each case had only one chance to be included in the validation set. The probability of all cases in the validation set was combined to perform the receiver operating characteristic curve (ROC) analysis, and the area under the curve (AUC) was calculated.

In addition to the radiomics analysis, we also built models using the clinical characteristics and the imaging features determined by visual reading, by following a similar process for feature selection and 10-fold crossvalidation. All clinical/imaging parameters were evaluated using a random forest algorithm. Then the features with the highest significance were selected to build the classification model. Random forest algorithms were utilized *via* Bootstrap-aggregated decision trees to evaluate the importance of these features in differentiating the high vs. low VEGF and wild-type vs. mutant p53 groups. A measurement of the feature significance can be assessed as the loss of accuracy after this feature was removed. The features were sorted based on their importance scores, and then, according to the ranking, the top 1, 2, 3,… features were selected to build the diagnostic model by using logistic regression. The discrimination accuracy was evaluated by the ROC analysis using 10-fold stratified crossvalidation. This process was repeated many times using a different combination of selected imaging or clinical features (1, 2, 3,…), and the results were used to find the best model according to the highest AUC. After the features included in the best model were decided, they were used to build a final diagnostic classifier with logistic regression, and the accuracy was evaluated in the entire dataset.

Lastly, a combined logistic regression model was built by using the selected clinical/imaging variables and the radiomics scores, which were evaluated using ROC.

### Statistical Analysis

For multivariate analysis of the importance of the two biomarkers for survival outcomes, we used a Cox regression model, which was performed using R 3.6.3 software (The R Foundation for Statistical Computing, Vienna, Austria) based on the patient’s outcome data, including PFS and p53/VEGF expression results. PFS was defined as the time between the date of surgery and the date of confirmed disease progression or death. PFS was censored at the date of death from other causes or the date of the last follow-up visit for progression-free patients. Progression was determined by the imaging evidence of the postoperative follow-up that showed an emerging soft tissue mass in the operation area, and pathological puncture was performed if necessary. Results were reported as hazard ratios (HR) with 95% confidence intervals (95% CI). Other statistical analyses were performed using SPSS version 18.0 (SPSS, Chicago, IL, USA). For clinical characteristics and general imaging features between different biomarker groups, the significance of each variable was tested by using the independent samples *t*-test, *χ* ^2^ test, or Mann–Whitney *U* test, depending on the data type. The ROC analysis was used to evaluate the performance of three different models, and the AUC was calculated and compared using the DeLong test. A 2-sided *p*-value of <0.05 was regarded as statistically significant.

## Results

### Patient Characteristics

The present study included 80 patients, of whom 43.75% (35 patients) were men and 56.25% were women (45 patients). Based on the IHC results, 31 had grades 0–1 (low VEGF) and 49 had grades 2–3 (high VEGF) expression; 68 had wild-type p53 and 12 had mutant p53. The clinical and imaging characteristics of patients in different VEGF and p53 groups are listed in **Table 1**.

**Table 1:** Clinical and imaging characteristics in high vs. low VEGF groups and wild-type vs. mutant p53 groups.

Parameter	High VEGF (*N* = 49)	Low VEGF (*N* = 31)	*p*-value	Wild-type p53 (*N* = 68)	Mutant p53 (*N* = 12)	*p*-value
**Clinical characteristics**						
Age	33.3 ± 13.3	32.2 ± 10.7	0.701	32.6 ± 12.0	34.3 ± 14.0	0.694
VAS score	5.9 ± 1.6	6.4 ± 1.4	0.126	6.1 ± 1.6	6.1 ± 1.2	0.962
SINS score	12.2 ± 2.0	9.9 ± 2.0	<0.001^*^	11.0 ± 2.2	12.8 ± 2.4	0.030^*^
Symptom duration (months)	12.9 ± 6.8	12.6 ± 4.1	0.769	12.0 ± 4.9	17.4 ± 8.4	0.05
Intraoperative bleeding vol (ml)	1125 ± 565	574 ± 303	<0.001^*^	843 ± 452	1,300 ± 854	0.095
**Spinal stability**			<0.001^*^			0.013^*^
0: Stable	24 (49.0%)	28 (90.3%)		48 (70.6%)	4 (33.3%)	
1: Unstable	25 (51.0%)	3 (9.7%)		20 (29.4%)	8 (66.7%)	
**Enneking stage**			0.910			0.017^*^
1	31 (63.3%)	20 (64.5%)		47 (69.1%)	4 (33.3%)	
2	18 (36.7%)	11 (35.5%)		21 (30.9%)	8 (66.7%)	
**Imaging characteristics**						
**Lesion location**			0.242			0.642
Cervical	18 (36.7%)	8 (25.8%)		22 (32.4%)	4(33.3%)	
Thoracic	20 (40.8%)	13 (41.9%)		27 (39.7%)	6 (50.0%)	
Lumbar	5 (10.2%)	8 (25.8%)		11 (16.2%)	2 (16.7%)	
Sacral	6 (12.3%)	2 (6.5%)		8 (11.7%)	0 (0.0%)	
**Position**			0.386			0.861
Vertebral body	43 (87.8%)	25 (80.6%)		58 (85.3%)	10 (83.3%)	
Vertebral arch	6 (12.2%)	6 (19.4%)		10 (14.7%)	2 (16.7%)	
**Vertebral compression**			0.981			0.741
0%	15 (30.6%)	10 (32.3%)		22 (32.4%)	3 (25.0%)	
≤50%	20 (40.8%)	12 (38.7%)		26 (38.2%)	6 (50.0%)	
>50%	14 (28.6%)	9 (29.0%)		20 (29.4%)	3 (25.0%)	
**Lesion boundary**			0.636			0.044*
Clear	47 (95.9%)	29 (93.5%)		66 (97.1%)	10 (83.3%)	
Unclear	2 (4.1%)	2 (6.5%)		2 (2.9%)	2 (16.7%)	
**Residual bone crest**			0.779			0.443
Yes	19 (38.8%)	13 (41.9%)		26 (38.2%)	6 (50.0%)	
No	30 (61.2%)	18 (58.1%)		42 (61.8%)	6 (50.0%)	
**“Soap bubble-like” sign**			0.815			0.292
Yes	45 (91.8%)	28 (90.3%)		63 (92.6%)	10 (83.3%)	
No	4 (8.2%)	3 (9.7%)		5 (7.4%)	2 (16.7%)	
**CT Hounsfield value**	48.1 ± 9.5	47.5 ± 10.0	0.795	47.8 ± 9.9	48.2 ± 8.3	0.905
**Largest diameter**	4.9 ± 1.7	5.3 ± 2.1	0.310	5.1 ± 1.9	4.8 ± 1.1	0.428

^*^p-value <0.05; VAS, Visual Analog Scale; SINS, Spinal Instability Neoplastic Score.

### Multivariate Analysis of Prognostic Factors for PFS

As shown in **Figure 3**, the multivariable Cox regression analysis showed that p53 and VEGF were significantly associated with PFS (*p* < 0.001). The results showed that the mutant p53 group had a significantly poorer PFS than the wild-type group (HR: 4.231; 95% CI: 1.663–10.768; *p* < 0.01). Patients with high VEGF expression also had a worse PFS than patients with low VEGF expression (HR: 2.891; 95% CI: 1.053–7.935; *p* = 0.039). The Cox proportional risk regression model confirmed that p53 and VEGF are independent prognostic factors for spinal GCTB.

### Relationship Between p53/VEGF Expression and Clinical Characteristics and General Imaging Features

The SINS score, or the dichotomized spinal stability, showed significant differences between high and low VEGF groups, all with *p* < 0.001 in univariate analysis. When using the single parameter to construct ROC, the AUC was 0.781 (95% CI: 0.676–0.886) for the SINS score. The SINS score (or the dichotomized stability) and the Enneking stage were significantly different between patients with wild-type and mutant p53. The AUC was 0.737 (95% CI: 0.562–0.913) for the SINS score and 0.679 (95% CI: 0.511–0.847) for the Enneking stage. For the general imaging features, none was significantly different between the two VEGF groups, and only one variable, the boundary of the lesion, showed a marginal difference between wild-type and mutant p53 (*p* = 0.044).

### Development of Radiomics, Clinical, and Combined Models

**Figure 5:**
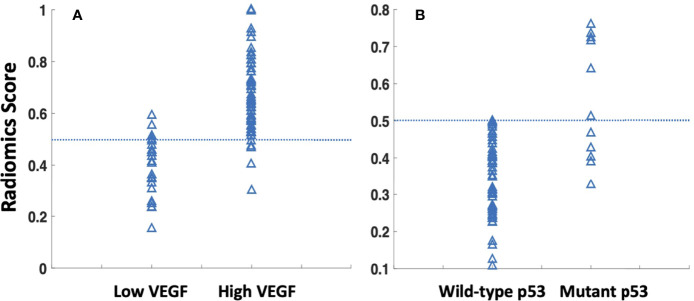
The radiomics scores were calculated using the developed radiomics models for all cases, classified using 0.5 as the threshold. **(A)** For prediction of low vs. high VEGF groups, showing 27 true low VEGF, 4 false low VEGF, 44 true high VEGF, and 5 false high VEGF. **(B)** For wild-type vs. mutant p53 groups, showing 68 true WT-p53, 0 false WT-p53, 7 true mutant p53, and 5 false mutant p53.

**Figure 6:**
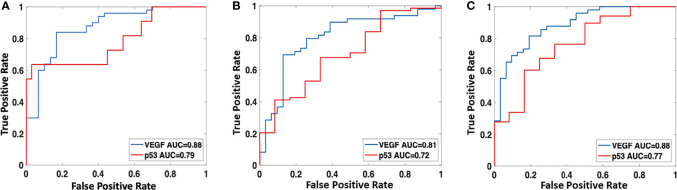
The ROC curves were constructed by using the best models developed using **(A)** radiomics analysis; **(B)** clinical and imaging variables; and **(C)** combined radiomics scores and clinical/imaging variables.

The radiomics model was built using features selected by the sequential SVM method. For high vs. low VEGF, 4 features were selected: major axis length, GLCM_contrast, GLCM_IDMN, and GLRLM_gray level variance. The best model had an AUC of 0.88 and an accuracy of 89% when using the radiomics score of 0.5 as the classification threshold. For wild-type vs. mutant p53, three features were selected: GLCM_entropy, GLDM_small dependence emphasis, and the surface-to-volume ratio. The best model had an AUC of 0.79 and an accuracy of 95%. The radiomics scores calculated from the models built for VEGF and p53 are shown in **Figure 5**. The ROC curves are shown in **Figure 6**. Abbreviations for features are shown in **Supplementary Part 3**.

The best conventional model built by considering the clinical and imaging variables yielded an AUC of 0.81 for VEGF and 0.72 for p53. The selected features, in sequence, were SINS and VAS for VEGF and SINS and Enneking stage for p53. The radiomics score and the selected clinical/imaging variables were then combined to build another model by using logistic regression. When using the radiomics score with SINS and VAS for VEGF, the achieved AUC was 0.88. When using the radiomics score with SINS and Enneking stage for p53, the achieved AUC was 0.77. The AUC for these models was not significantly different using the DeLong test. The classification sensitivity, specificity, accuracy, and AUC are summarized in **Table 2**.

**Table 2:** The classification results of three models built using radiomics analysis, clinical and imaging characteristics, and the combined model (high VEGF and mutant p53 as positive).

		Sensitivity	Specificity	Accuracy	AUC
High vs. low VEGF^*^	Radiomics analysis	44/49 (90%)	27/31 (87%)	71/80 (89%)	0.88
	Clinical + imaging	44/49 (90%)	15/31 (48%)	64/80 (80%)	0.81
	Combined model	41/49 (84%)	27/31 (87%)	68/80 (85%)	0.88
**Mutant vs. Wild-type p53**	Radiomics analysis	7/12 (58%)	68/68 (100%)	75/80 (94%)	0.79
	Clinical + imaging	5/12 (42%)	61/68 (90%)	66/80 (83%)	0.72
	Combined model	7/12 (58%)	64/68 (94%)	71/80 (89%)	0.77

^*^VEGF, vascular endothelial growth factor. Low group includes grades 0 and 1 and high group includes grades 2 and 3.

## Discussion

There were two objectives in this study: first to evaluate the prognostic value of two tumor markers, p53 and VEGF, in the PFS of spinal GCTB, and second to build models based on the pre-perative CT radiomics features and clinical variables for the classification of the VEGF and p53 status. The Cox proportional hazards regression model confirmed the prognostic role of p53 and VEGF. The models may help to predict the biological behavior of the tumor and provide preoperative risk stratification information to aid in the selection of appropriate treatments. The analysis based on imaging of the entire tumor may help overcome the limitations of preoperative tissue sampling. Three models were built using (1) radiomics features, (2) clinical + conventional imaging variables, and (3) combined radiomics scores and selected clinical variables. The AUC of the three models for classifying high vs. low VEGF were 0.88, 0.81, and 0.88, respectively, and for wild-type vs. mutant p53 were 0.79, 0.72, and 0.77, respectively. The results support that the clinical variables and radiomics features contained in preoperative CT were related to IHC biomarkers.

In the era of precision medicine, molecular markers have been established as important diagnostic and prognostic markers in clinical decision-making (41). Our study found that high VEGF status was associated with worse postoperative survival of patients. Several studies have shown that high levels of p53/VEGF expression are associated with high recurrence rates (14, 15, 42), and therapies targeting these two biomarkers are under research or in clinical trials (13, 43–45). VEGF is one of the most important growth factors for the regulation of vascular development and angiogenesis (18). The interaction between endothelial cells and bone cells is essential for bone formation during bone remodeling and repair (46, 47). According to this mechanism, interferon is used for the treatment of GCTB and has shown some promising efficacy (42, 43, 45). The pharmacologic treatment using interferon may provide an option for unresectable, recurring, and metastatic GCTB that failed the bisphosphonates or denosumab or could not be continued due to complications.

As for p53, mutant p53 is a well-known poor prognostic indicator for many tumors, including sarcoma (11, 12). Previous studies in GCTB have also shown that p53 is an important prognostic marker for predicting local recurrence and lung metastasis in GCTB (48–50). Yalcinkaya et al. reported a significant relationship between p53 expression and local recurrence (*p* = 0.022) (49). In patients with lung metastases, weakly positive staining was found in GCTB of the tibia and vertebra. However, there are currently no long-term follow-up survival studies after receiving the same surgical procedure using the TES for spinal GCTB to evaluate the specific correlation between p53 status and the prognosis of patients. Our findings provide evidence for this patient cohort through longer-term clinical follow-up. Although many of the potential therapies are at the preclinical testing stage, they may offer a new approach for osteosarcoma treatment based on p53 targeting in the future (44).

Although the IHC biomarkers are known to be important, the assessment will require high-quality tissue specimens for immunohistochemical staining. For preoperative evaluation, biopsy needle puncture may not provide a sufficient amount of tumorous tissue for analysis, and the results might also be affected by the tumor heterogeneity. As shown in our results, imaging may provide information associated with the IHC biomarkers, which can be acquired noninvasively and with a very high spatial resolution covering the entire tumor.

Several clinical variables were considered in the analysis. Among them, the intraoperative bleeding volume and the SINS score were found to be significantly different between the high VEGF and low VEGF groups. SINS was also significantly different between wild-type and mutant p53. The results showed that the spinal instability was associated with the expression of VEGF and p53 as a poor prognostic indicator. This is consistent with the role of SINS related to survival time reported in the literature (51). The amount of bleeding during surgery was greater in the high VEGF group, which was anticipated with the association between VEGF and the abundance of blood supply. The VEGF results may help the orthopedic surgeon estimate the degree of bleeding before surgery and, if necessary, to perform preoperative embolization. However, as a parameter that can only be obtained after surgery, the amount of bleeding is not included in our prediction model. We also found that the Enneking staging was significantly related to the p53 status, which was consistent with previous reports about the role of Enneking staging in planning surgery and adjuvant therapy for bone tumors and tumor-like bone lesions (52).

Imaging has always been an important examination for preoperative tumor evaluation, but most of the previous studies on GCTB focused on tumors of the extremities (53–56), which led to many findings of indicators not applicable to the spinal tumors, such as the distance between the edge of the tumor and joint surface, “paintbrush borders” sign, destruction of posterior cortical bone, and depth of local tumor cell infiltration. Although the majority of GCTB lesions are located in the metaphysis and epiphyses of the long tubular bones, approximately one-third of tumors are located in the axial skeleton. Our study included some features of spinal GCTB for evaluation but did not find the significance of specific imaging indicators. In this study, the unclear boundary of the lesion was the only feature related to mutant p53, which was consistent with the finding of other MRI studies showing that lesions with unclear boundaries had more aggressive biological behaviors (56, 57).

Radiomics analysis is a high-throughput method to extract a large number of features from radiographic images, which has been shown as a promising method for the diagnosis and further characterization of tumors (58). In this study, we used the SVM with Gaussian kernel for selecting important radiomics features and for building the classification models (59). The kernel in SVM works as a transformation that maps input parameters into a different feature space where the transformed data can be divided more obviously to reach a higher accuracy (60, 61). Other classification models, such as logistic regression and decision trees, work in the original feature space, so less flexible. Meanwhile, the cost function of SVM allows defining margins between different groups. This can improve the robustness of the model and avoid overfitting during the training process. For studies with a limited case number, SVM is considered the best option to balance the variance and bias of the input data (60, 61). In this study, CT imaging was analyzed because it was cost-effective and considered the most commonly used for the management of bone tumors in clinical practice.

Radiomics analysis has also been applied to predict the status of VEGF (angiogenesis) and p53 in various cancers in the literature. Wang et al. investigated the value of a radiomics model based on dynamic contrast-enhanced magnetic resonance imaging (DCE-MRI) and diffusion-weighted imaging (DWI) in estimating the isocitrate dehydrogenase 1 (IDH1) mutation and angiogenesis in gliomas, which suggested that the SVM model showed good performance for predicting the VEGF expression (validation group, AUC = 0.919) (62). Sun et al. developed a machine-learning model for predicting VEGF status in patients with diffuse gliomas, and the AUC was 74.1% in the training group and 70.2% in the validation group (63). Other studies have also applied radiomics based on different imaging techniques to predict the expression status of p53 in epithelial ovarian cancer (64), endometrial carcinoma (65), esophageal squamous cell carcinoma (66), and breast ductal carcinoma (67).

The major limitation was the small sample size identified from a retrospective clinical database. GCTB in the spine was rare, and even in our tertiary hospital specializing in bone diseases, we had to review the records over 10 years to find these cases. Also, to control for the confounding factors of different surgical methods on the progression-free survival, only patients receiving the TES were eligible for this study, which further limited the case number and the difficulty to identify an independent dataset for validation. In our analysis, we applied the 10-fold crossvalidation, so the final model has gone through rigorous validations. Another inherent limitation was the unbalanced dataset for p53 because the mutant p53 was rare. Therefore, in the analysis, we were focusing on the ROC, not the accuracy (68/80 = 85% accuracy, if assuming all cases were wild-type). In addition, some important variables were not detailed in this study, such as age, SINS, Enneking stage, etc. To further refine the description, we performed a Cox regression analysis based on these factors, which is shown in **Supplementary Part 4**. Nonetheless, we believe the results from this difficult-to-obtain dataset can contribute new knowledge to the management of spinal GCTB. The developed models can be applied to prospective patients for further validation.

In summary, our study demonstrates that VEGF and p53 are potential biomarkers for progression-free survival prediction of spinal GCTB patients. Meanwhile, we have shown that radiomics features extracted from preoperative CT imaging can be used to build models for the classification of VEGF and p53 status in spinal GCTB. The capability to predict the aggressive biological phenotype in spinal GCTB based on preoperative information may help to improve management, including choosing optimal treatment strategies and better surveillance protocols.

## Data Availability Statement

The raw data supporting the conclusions of this article will be made available by the authors, without undue reservation.

## Ethics Statement

The studies involving human participants were reviewed and approved by Peking University Third Hospital Medical Science Research Ethics Committee. Written informed consent for participation was not required for this study in accordance with the national legislation and the institutional requirements.

## Author Contributions

Study management and guidance: NL and M-YS. Study design: NL and QW. Clinical data acquisition and analysis: QW and YZ. Imaging data acquisition and analysis: QW, EZ, and XX. Experimental studies: QW, EZ, and YC. Manuscript preparation: QW, YZ, KN, and HY. Manuscript review: NL and MS. All authors listed have made a substantial, direct, and intellectual contribution to the work and approved it for publication.

## Funding

This study received funding from the National Natural Science Foundation of China (81971578, 81871326) and the Key Clinical Projects of the Peking University Third Hospital (BYSY2018007).

## Conflict of Interest

The authors declare that the research was conducted in the absence of any commercial or financial relationships that could be construed as a potential conflict of interest.

## Publisher’s Note

All claims expressed in this article are solely those of the authors and do not necessarily represent those of their affiliated organizations, or those of the publisher, the editors and the reviewers. Any product that may be evaluated in this article, or claim that may be made by its manufacturer, is not guaranteed or endorsed by the publisher.
