# Supplementary material for: A Multiparametric Method Based on Clinical and CT-Based Radiomics to Predict the Expression of p53 and VEGF in Patients With Spinal Giant Cell Tumor of Bone
Source: Front Oncol. 2022 Jun 21;12:894696. doi: 10.3389/fonc.2022.894696 (PMC9253421; doi:10.3389/fonc.2022.894696)
Supplement: Supplementary file 1 [file DataSheet_1.pdf]

Supplementary Material

Supplementary Part 1

Relevant Clinical Rating Scale: Although a detailed discussion of the Spinal Instability Neoplastic Score (SINS), the Visual Analog Scale (VAS) and Enneking stage is beyond the scope of this manuscript, a brief description is worthwhile.

Supplementary Table 1: The Spinal Instability Neoplastic Score (SINS) for Assessing Mechanical Instability in Spinal Giant Cell Tumor of Bone

Component	SINS
**Location**	
Junctional (0-C2; C7-T2; T11-L1; L5-S1)	3
Mobile spine (C3-C6; L2-L4)	2
Semirigid (T3-T10)	1
Rigid (S2-S5)	0
**Pain**	
Mechanical	3
Oncologic	2
Pain-free lesion	1
**Bone lesion**	
Lytic	2
Mixed (blastic/lytic)	1
Blastic	0
**Vertebral body collapse**	
>50% collapse	3
<50% collapse	2
No collapse with >50% body involvement	1
None of the above	0
**Radiographic alignment**	
Subluxation/translation	4
Deformity (kyphosis/scoliosis)	2
Normal	0
**Posterolateral involvement**	
Bilateral	3
Unilateral	1
None of the above	0

Note: The Spinal Instability Neoplastic Score (SINS) was developed to assess the degree of spinal (in)stability in a standardized way. SINS could enhance the uniform reporting of spinal neoplastic-related instability in scientific studies.

Supplementary Figure 1. Assessing Pain Intensity with the Visual Analog Scale (VAS)

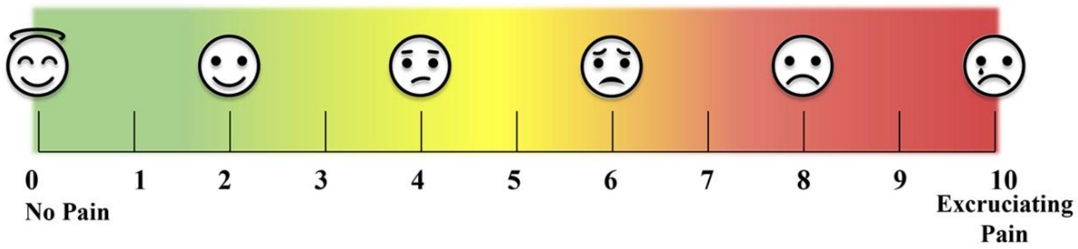

Note: The Visual Analogue Scale (VAS) is a popular tool for the measurement of pain. It consists of a line usually 100 mm in length, with anchor descriptors such as (in the pain context) “no pain” and “Excruciating Pain” depicted in Fig.1. The colors and facial expressions along the line can make it easier for patients to indicate their pain levels.

Supplementary Table 2: Enneking staging for musculoskeletal tumors based on surgical grade, local extent, and presence or absence of metastasis.

Stage	Grade	Site	Metastasis
IA	Low (G1)	Intracompartmental (T1)	No metastasis (M0)
IB	Low (G2)	Extracompartmental (T2)	No metastasis (M0)
IIA	High (G2)	Intracompartmental (T1)	No metastasis (M0)
IIB	High (G2)	Extracompartmental (T2)	No metastasis (M0)
III	Any (G)	Any (T)	Regional or distant metastasis (M1)

Note: The Enneking classification is based on the interrelationship of the biologic grade (G), the local extent of the tumor (T), and the presence of metastases (M). The tumors are divided into 3 grades according to their biologic behavior, with G0 denoting benign tumors, G1 low-grade malignant tumors, and G2 high-grade malignant tumors. The local extent of the tumor varies from intracapsular (T0), through intracompartmental (T1), to extracompartmental (T2). Metastases may be absent (M0) or present (M1). These 3 factors combine to give the Enneking stages.
