# Supplementary material for: A Multiparametric Method Based on Clinical and CT-Based Radiomics to Predict the Expression of p53 and VEGF in Patients With Spinal Giant Cell Tumor of Bone
Source: Front Oncol. 2022 Jun 21;12:894696. doi: 10.3389/fonc.2022.894696 (PMC9253421; doi:10.3389/fonc.2022.894696)
Supplement: Supplementary file 2 [file DataSheet_2.pdf]

Supplementary Materials: The list of 107 PyRadiomics Features

The 107 imaging features were extracted using the most commonly used radiomics analysis software, PyRadiomics. http://www.radiomics.io/pyradiomics.html

The table below lists all 107 features, which are obtained using several different algorithms, including First-Order (N=18); Shape (N=14); GLCM (N=24); GLSZM (N=16); GLRLM (N=16); GLDM (N=14); and NGTDM (N=5). The basic definition and terminology used in each algorithm are explained, and the explicit mathematical equation for each feature is given at the end of this supplementary.

Supplementary Table 1: List of 107 PyRadiomics Features

**1st Order (N=18)**	**Shape (N=14)**	**GLCM (N=24)**	**GLSZM (N=16)**	**GLRLM (N=16)**	**GLDM (N=14)**
Energy	Mesh Volume	Autocorrelation	SAE	SRE	SDE
Entropy	Voxel Volume	Joint Average	LAE	LRE	LDE
Minimum	Surface Area	Cluster Prominence	GLN	GLN	GLN
10th percentile	Surface Area/Volume ratio	Cluster Shade	GLNN	GLNN	DN
90th percentile	Sphericity	Cluster Tendency	SZN	RLN	DNN
Maximum	Max 3D diameter	Contrast	SZNN	RLNN	GLV
Mean	Max 2D diameter (Slice)	Correlation	ZP	RP	DV
Median	Max 2D diameter (Column)	Difference Average	GLV	GLV	DE
Interquartile Range	Max 2D diameter (Row)	Difference Entropy	ZV	RV	LGLE
Range	Major Axis Length	Difference Variance	ZE	RE	HGLE
MAD	Minor Axis Length	Joint Energy	LGLZE	LGLRE	SDLGLE
rMAD	Least Axis Length	Joint Entropy	HGLZE	HGLRE	SDHGLE
RMS	Elongation	IMC1	SALGLE	SRLGLE	LDLGLE
Std	Flatness	IMC2	SAHGLE	SRHGLE	LDHGLE
Skewness		IDM	LALGLE	LRLGLE	
Kurtosis		MCC	LAHGLE	LRHGLE	
Variance		IDMN			
Uniformity		ID			
	**NGTDM (N=5)**	IDN			
	coarseness	Inverse Variance			
	contrast	Sum Average			
	busyness	Sum Entropy			
	complexity	Max Probability			
	strength	Sum of Squares			

First Order Features, N=18

First-order statistics describe the distribution of voxel intensities within the image region defined by the mask through commonly used and basic metrics.
Let:

- $\mathbf{X}$ be a set of Np voxels included in the ROI
- $\mathbf{P}(i)$ be the first order histogram with Ng discrete intensity levels, where Ng is the number of non-zero bins.
- $p(i)$ be the normalized first order histogram and equal to $\mathbf{P}(i)Np$

1. Energy

$$\text{energy} = \sum_{i=1}^{N_p} (\mathrm{X}(i) + c)^2$$

Here, c is optional value, which shifts the intensities to prevent negative values in $\mathbf{X}$. This ensures that voxels with the lowest gray values contribute the least to Energy, instead of voxels with gray level intensity closest to 0. Energy is a measure of the magnitude of voxel values in an image. A larger value implies a greater sum of the squares of these values.

2. Entropy

$$\text{entropy} = -\sum_{i=1}^{N_g} p(i)\log_2 (p(i) + \epsilon)$$

Here, ϵ is an arbitrarily small positive number (≈2.2×10−16).
Entropy specifies the uncertainty/randomness in the image values. It measures the average amount of information required to encode the image values.

3. Minimum

minimum=min($\mathbf{X}$)

4. 10th percentile The 10th percentile of $\mathbf{X}$

5. 90th percentile The 90th percentile of $\mathbf{X}$

6. Maximum The maximum gray level intensity within the ROI.

maximum=max($\mathbf{X}$)

7. Mean The average gray level intensity within the ROI.

$$\text{mean} = \frac{1}{N_p}\sum_{i=1}^{N_p} \mathrm{X}(i)$$

8. Median The median gray level intensity within the ROI.

9. Interquartile Range

interquartile range=$\mathbf{P}$75−$\mathbf{P}$25

Here $\mathbf{P}$25 and $\mathbf{P}$75 are the 25th and 75th percentile of the image array, respectively.

10. Range The range of gray values in the ROI.

range=max($\mathbf{X}$)−min($\mathbf{X}$)

11. Mean Absolute Deviation (MAD)

$$\text{MAD} = \frac{1}{N_p}\sum_{i=1}^{N_p} |\mathrm{X}(i) - \overline{X}|$$

Mean Absolute Deviation is the mean distance of all intensity values from the Mean Value of the image array.

12. Robust Mean Absolute Deviation (rMAD)

$$\text{rMAD} = \frac{1}{N_{10-90}} \sum_{i=1}^{N_{10-90}} |\text{X}_{10-90}(i) - \bar{X}_{10-90}|$$

Robust Mean Absolute Deviation is the mean distance of all intensity values from the Mean Value calculated on the subset of image array with gray levels in between, or equal to the 10th and 90th percentile.

13. Root Mean Squared (RMS)

$$\text{RMS} = \sqrt{\frac{1}{N_p} \sum_{i=1}^{N_p} (\text{X}(i) + c)^2}$$

Here, cc is optional value, which shifts the intensities to prevent negative values in $\mathbf{X}$. This ensures that voxels with the lowest gray values contribute the least to RMS, instead of voxels with gray level intensity closest to 0.

RMS is the square-root of the mean of all the squared intensity values. It is another measure of the magnitude of the image values. This feature is volume-confounded, a larger value of cc increases the effect of volume-confounding.

14. Standard Deviation

$$\text{standard deviation} = \sqrt{\frac{1}{N_p} \sum_{i=1}^{N_p} (\text{X}(i) - \bar{X})^2}$$

standard

Standard Deviation measures the amount of variation or dispersion from the Mean Value.

15. Skewness

$$\text{skewness} = \frac{\mu_3}{\sigma^3} = \frac{\frac{1}{N_p} \sum_{i=1}^{N_p} (\text{X}(i) - \bar{X})^3}{\left(\sqrt{\frac{1}{N_p} \sum_{i=1}^{N_p} (\text{X}(i) - \bar{X})^2}\right)^3}$$

Where $\mu 3$/μ3 is the 3rd central moment.

Skewness measures the asymmetry of the distribution of values about the Mean value. Depending on where the tail is elongated and the mass of the distribution is concentrated, this value can be positive or negative.

16. Kurtosis

$$\text{skewness} = \frac{\mu_3}{\sigma^3} = \frac{\frac{1}{N_p} \sum_{i=1}^{N_p} (\text{X}(i) - \bar{X})^3}{\left(\sqrt{\frac{1}{N_p} \sum_{i=1}^{N_p} (\text{X}(i) - \bar{X})^2}\right)^3}$$

Where $\mu 4$/μ4 is the 4th central moment.

Kurtosis is a measure of the 'peakedness' of the distribution of values in the image ROI. A higher kurtosis implies that the mass of the distribution is concentrated towards the tail(s) rather than towards the mean. A lower kurtosis implies the reverse: that the mass of the distribution is concentrated towards a spike near the Mean value.

17. Variance

$$\text{variance} = \frac{1}{N_p} \sum_{i=1}^{N_p} (\text{X}(i) - \bar{X})^2$$

Variance is the mean of the squared distances of each intensity value from the Mean value. This is a measure of the spread of the distribution about the mean. By definition, variance=σ^2

18. Uniformity

$$\text{uniformity} = \sum_{i=1}^{N_g} p(i)^2$$

Uniformity is a measure of the sum of the squares of each intensity value. This is a measure of the homogeneity of the image array, where a greater uniformity implies a greater homogeneity or a smaller range of discrete intensity values.

Shape Features (3D), N=14

In this group of features we included descriptors of the three-dimensional size and shape of the ROI. These features are independent from the gray level intensity distribution in the ROI and are therefore only calculated on the non-derived image and mask. Unless otherwise specified, features are derived from the approximated shape defined by the triangle mesh. To build this mesh, vertices (points) are first defined as points halfway on an edge between a voxel included in the ROI and one outside the ROI. By connecting these vertices a mesh of connected triangles is obtained, with each triangle defined by 3 adjacent vertices, which shares each side with exactly one other triangle. This mesh is generated using a marching cubes algorithm. In this algorithm, a 2x2 cube is moved through the mask space. For each position, the corners of the cube are then marked 'segmented' (1) or 'not segmented' (0). Treating the corners as specific bits in a binary number, a unique cube-index is obtained (0-255). This index is then used to determine which triangles are present in the cube, which are defined in a lookup table. These triangles are defined in such a way, that the normal (obtained from the cross product of vectors describing 2 out of 3 edges) are always oriented in the same direction. Let:

- Nv represent the number of voxels included in the ROI
- Nf represent the number of faces (triangles) defining the Mesh.
- V the volume of the mesh in mm^3,
- AA the surface area of the mesh in mm^2

1. Mesh Volume

$$V_i = \frac{Oa_i \cdot (Ob_i \times Oc_i)}{6} \text{ (1)}$$

$$V = \sum_{i=1}^{N_f} V_i \text{ (2)}$$

The volume of the ROI V is calculated from the triangle mesh of the ROI. For each face i in the mesh, defined by points ai,bi and ci, the (signed) volume Vf of the tetrahedron defined by that face and the origin of the image (O) is calculated. The sign of the volume is determined by the sign of the normal, which must be consistently defined as either facing outward or inward of the ROI.

2. Voxel Volume

$$V_{voxel} = \sum_{k=1}^{N_v} V_k$$

The volume of the ROI $Vvoxel$ is approximated by multiplying the number of voxels in the ROI by the volume of a single voxel Vk. This is a less precise approximation of the volume and is not used in subsequent features. This feature does not make use of the mesh and is not used in calculation of other shape features.

3. Surface Area

$$A_i = \frac{1}{2}|\text{a}_i\text{b}_i \times \text{a}_i\text{c}_i| \text{ (1)}$$

$$A = \sum_{i=1}^{N_f} A_i \text{ (2)}$$

where: aibi and aici are edges of the ith triangle in the mesh, formed by vertices a$i$ai, b$i$bi and c$i$ci.

4. Surface Area to Volume ratio

$$\text{surface to volume ratio}=A/V$$

Here, a lower value indicates a more compact (sphere-like) shape. This feature is not dimensionless, and is therefore (partly) dependent on the volume of the ROI.

5. Sphericity

$$\text{sphericity} = \frac{\sqrt[3]{36\pi V^2}}{A}$$

Sphericity is a measure of the roundness of the shape of the tumor region relative to a sphere. It is a dimensionless measure, independent of scale and orientation. The value range is 0<$sphericity$≤10<sphericity≤1, where a value of 1 indicates a perfect sphere (a sphere has the smallest possible surface area for a given volume, compared to other solids).

6. Maximum 3D diameter

Maximum 3D diameter is defined as the largest pairwise Euclidean distance between tumor surface mesh vertices.

7. Maximum 2D diameter (Slice)

Maximum 2D diameter (Slice) is defined as the largest pairwise Euclidean distance between tumor surface mesh vertices in the row-column (generally the axial) plane.

8. Maximum 2D diameter (Column)

Maximum 2D diameter (Column) is defined as the largest pairwise Euclidean distance between tumor surface mesh vertices in the row-slice (usually the coronal) plane.

9. Maximum 2D diameter (Row)

Maximum 2D diameter (Row) is defined as the largest pairwise Euclidean distance between tumor surface mesh vertices in the column-slice (usually the sagittal) plane.

10. Major Axis Length

$$\text{major axis} = 4\sqrt{\lambda_{major}}$$

This feature yield the largest axis length of the ROI-enclosing ellipsoid and is calculated using the largest principal component $\lambda major$.

The principal component analysis is performed using the physical coordinates of the voxel centers defining the ROI. It therefore takes spacing into account, but does not make use of the shape mesh.

11. Minor Axis Length

$$\text{minor axis} = 4\sqrt{\lambda_{minor}}$$

This feature yield the second-largest axis length of the ROI-enclosing ellipsoid and is calculated using the largest principal component $\lambda minor$.

The principal component analysis is performed using the physical coordinates of the voxel centers defining the ROI. It therefore takes spacing into account, but does not make use of the shape mesh.

12. Least Axis Length

$$\text{least axis} = 4\sqrt{\lambda_{least}}$$

This feature yield the smallest axis length of the ROI-enclosing ellipsoid and is calculated using the largest principal component $\lambda least$ In case of a 2D segmentation, this value will be 0.

13. Elongation

Elongation shows the relationship between the two largest principal components in the ROI shape. For computational reasons, this feature is defined as the inverse of true elongation.

$$\text{elongation} = \sqrt{\frac{\lambda_{minor}}{\lambda_{major}}}$$

Here, λmajor and λminor are the lengths of the largest and second largest principal component axes. The values range between 1 (where the cross section through the first and second largest principal moments is circle-like (non-elongated)) and 0 (where the object is a maximally elongated: i.e. a 1 dimensional line).

14. Flatness

Flatness shows the relationship between the largest and smallest principal components in the ROI shape. For computational reasons, this feature is defined as the inverse of true flatness.

$$\text{flatness} = \sqrt{\frac{\lambda_{least}}{\lambda_{major}}}$$

Here, λmajor and λleast are the lengths of the largest and smallest principal component axes. The values range between 1 (non-flat, sphere-like) and 0 (a flat object, or single-slice segmentation).

Gray Level Co-occurrence Matrix (GLCM) Features, N=24

A Gray Level Co-occurrence Matrix (GLCM) of size $Ng \times Ng$ describes the second-order joint probability function of an image region constrained by the mask and is defined as $\mathbf{P}(i,j|\delta,\theta)$. The (i,j)th element of this matrix represents the number of times the combination of levels i and j occur in two pixels in the image, that are separated by a distance of δ pixels along angle θ. The distance δ from the center voxel is defined as the distance according to the infinity norm. For δ=1, this results in 2 neighbors for each of 13 angles in 3D (26-connectivity) and for δ=2 a 98-connectivity (49 unique angles).
As a two dimensional example, let the following matrix $\mathbf{I}$ represent a 5x5 image, having 5 discrete grey levels:

$$I = \begin{bmatrix} 1 & 2 & 5 & 2 & 3 \\ 3 & 2 & 1 & 3 & 1 \\ 1 & 3 & 5 & 5 & 2 \\ 1 & 1 & 1 & 1 & 2 \\ 1 & 2 & 4 & 3 & 5 \end{bmatrix}$$

For distance δ=1(considering pixels with a distance of 1 pixel from each other) and angle θ=0(horizontal plane, i.e. voxels to the left and right of the center voxel), the following symmetrical GLCM is obtained:

$$P = \begin{bmatrix} 6 & 4 & 3 & 0 & 0 \\ 4 & 0 & 2 & 1 & 3 \\ 3 & 2 & 0 & 1 & 2 \\ 0 & 1 & 1 & 0 & 0 \\ 0 & 3 & 2 & 0 & 2 \end{bmatrix}$$

Let:

- ϵ be an arbitrarily small positive number (≈2.2×10−16)
- $\mathbf{P}(i,j)$be the co-occurence matrix for an arbitrary δδ and θθ
- $p(i,j)$ be the normalized co-occurence matrix and equal to $\mathbf{P}(i,j) \sum \mathbf{P}(i,j)$
- Ng be the number of discrete intensity levels in the image
- $px(i)=\sum Ngj=1P(i,j)$ be the marginal row probabilities
- $py(j)=\sum Ngi=1P(i,j)$ be the marginal column probabilities
- μx be the mean gray level intensity of pxpx and defined as $\mu x=\sum i=1Ngpx(i)i$
- μy be the mean gray level intensity of py and defined as $\mu y=\sum j=1Ngpy(j)j$
- σx be the standard deviation of px
- σy be the standard deviation of py
- $px+y(k)=\sum Ngi=1\sum Ngj=1p(i,j)$, where $i+j=k$, and k=2,3,…,2Ng
- $px-y(k)=\sum Ngi=1\sum Ngj=1p(i,j)$, where $|i-j|=k$, and k=0,1,…,$Ng-1$
- $HX=-\sum Ngi=1px(i)\log2(px(i)+\epsilon)$ be the entropy of px
- $HY=-\sum Ngj=1py(j)\log2(py(j)+\epsilon)$ be the entropy of pypy
- $HXY=-\sum Ngi=1\sum Ngj=1p(i,j)\log2(p(i,j)+\epsilon)$ be the entropy of $p(i,j)$p(i,j)
- $HXY1=-\sum Ngi=1\sum Ngj=1p(i,j)\log2(px(i)py(j)+\epsilon)$
- $HXY2=-\sum Ngi=1\sum Ngj=1px(i)py(j)\log2(px(i)py(j)+\epsilon)$

By default, the value of a feature is calculated on the GLCM for each angle separately, after which the mean of these values is returned. If distance weighting is enabled, GLCM matrices are weighted by weighting factor W and then summed and normalized. Features are then calculated on the resultant matrix. Weighting factor W is calculated for the distance between neighboring voxels by:
$W = e - \|d\|2$, where d is the distance for the associated angle according to the norm specified in setting ‘weighting Norm’.

1. Autocorrelation

$$\text{autocorrelation} = \sum_{i=1}^{N_g} \sum_{j=1}^{N_g} p(i,j)ij$$

Autocorrelation is a measure of the magnitude of the fineness and coarseness of texture.

2. Joint Average

$$\text{joint average} = \mu_x = \sum_{i=1}^{N_g} \sum_{j=1}^{N_g} p(i,j)i$$

Returns the mean gray level intensity of the i distribution.

3. Cluster Prominence

$$\text{cluster prominence} = \sum_{i=1}^{N_g} \sum_{j=1}^{N_g} (i + j - \mu_x - \mu_y)^4 p(i,j)$$

Cluster Prominence is a measure of the skewness and asymmetry of the GLCM. A higher values implies more asymmetry about the mean while a lower value indicates a peak near the mean value and less variation about the mean.

4. Cluster Shade

$$\text{cluster shade} = \sum_{i=1}^{N_g} \sum_{j=1}^{N_g} (i + j - \mu_x - \mu_y)^3 p(i,j)$$

Cluster Shade is a measure of the skewness and uniformity of the GLCM. A higher cluster shade implies greater asymmetry about the mean.

5. Cluster Tendency

$$\text{cluster tendency} = \sum_{i=1}^{N_g} \sum_{j=1}^{N_g} (i + j - \mu_x - \mu_y)^2 p(i,j)$$

Cluster Tendency is a measure of groupings of voxels with similar gray-level values.

6. Contrast

$$\text{contrast} = \sum_{i=1}^{N_g} \sum_{j=1}^{N_g} (i - j)^2 p(i,j)$$

Contrast is a measure of the local intensity variation, favoring values away from the diagonal (i=j). A larger value correlates with a greater disparity in intensity values among neighboring voxels.

7. Correlation

$$\text{correlation} = \frac{\sum_{i=1}^{N_g} \sum_{j=1}^{N_g} p(i,j)ij - \mu_x \mu_y}{\sigma_x(i)\sigma_y(j)}$$

Correlation is a value between 0 (uncorrelated) and 1 (perfectly correlated) showing the linear dependency of gray level values to their respective voxels in the GLCM.

8. Difference Average

$$\text{difference average} = \sum_{k=0}^{N_g-1} k p_{x-y}(k)$$

Difference Average measures the relationship between occurrences of pairs with similar intensity values and occurrences of pairs with differing intensity values.

9. Difference Entropy

$$\text{difference entropy} = \sum_{k=0}^{N_g-1} p_{x-y}(k) \log_2 (p_{x-y}(k) + \epsilon)$$

Difference Entropy is a measure of the randomness/variability in neighborhood intensity value differences.

10. Difference Variance

$$\text{difference variance} = \sum_{k=0}^{N_g-1} (k - DA)^2 p_{x-y}(k)$$

Difference Variance is a measure of heterogeneity that places higher weights on differing intensity level pairs that deviate more from the mean.

11. Joint Energy

$$\text{joint energy} = \sum_{i=1}^{N_g} \sum_{j=1}^{N_g} (p(i,j))^2$$

Energy is a measure of homogeneous patterns in the image. A greater Energy implies that there are more instances of intensity value pairs in the image that neighbor each other at higher frequencies.

12. Joint Entropy

$$\text{joint energy} = \sum_{i=1}^{N_g} \sum_{j=1}^{N_g} (p(i,j))^2$$

Joint entropy is a measure of the randomness/variability in neighborhood intensity values.

13. Informational Measure of Correlation (IMC) 1

$$\text{IMC 1} = \frac{HXY - HXY1}{max\{HX, HY\}}$$

IMC1 assesses the correlation between the probability distributions of $i$iand jj(quantifying the complexity of the texture), using mutual information I(x, y)
 This reflects how this feature is defined in the original Haralick paper.
In the case where the distributions are independent, there is no mutual information and the result will therefore be 0. In the case of uniform distribution with complete dependence, mutual information will be equal to log2(Ng)l

Finally, $HXY-HXY1$ is divided by the maximum of the 2 marginal entropies, where in the latter case of complete dependence (not necessarily uniform; low complexity) it will result in $IMC1=-1$, as $HX=HY=I(i,j)$.

14. Informational Measure of Correlation (IMC) 2

$$\text{IMC 2} = \sqrt{1 - e^{-2(HXY2-HXY)}}$$

IMC2 also assesses the correlation between the probability distributions of i and j (quantifying the complexity of the texture). Of interest is to note that $HXY1=HXY2$ and that $HXY2-HXY\geq 0$ represents the mutual information of the 2 distributions. Therefore, the range of IMC2 = [0, 1), with 0 representing the case of 2 independent distributions (no mutual information) and the maximum value representing the case of 2 fully dependent and uniform distributions (maximal mutual information, equal to log2(Ng).

15. Inverse Difference Moment (IDM)

$$\text{IDM} = \sum_{k=0}^{N_g-1} \frac{p_{x-y}(k)}{1+k^2}$$

IDM is a measure of the local homogeneity of an image. IDM weights are the inverse of the Contrast weights (decreasing exponentially from the diagonal i=j in the GLCM).

16. Maximum Correlation Coefficient

$$\text{MCC} = \sqrt{\text{second largest eigenvalue of Q}}$$

$$Q(i,j) = \sum_{k=0}^{N_g} \frac{p(i,k)p(j,k)}{p_x(i)p_y(k)}$$

The Maximum Correlation Coefficient is a measure of complexity of the texture.
In case of a flat region, each GLCM matrix has shape (1, 1), resulting in just 1 eigenvalue. In this case, an arbitrary value of 1 is returned.

17. Inverse Difference Moment Normalized (IDMN)

$$\text{IDMN} = \sum_{k=0}^{N_g-1} \frac{p_{x-y}(k)}{1+\left(\frac{k^2}{N_g^2}\right)}$$

IDMN (inverse difference moment normalized) is a measure of the local homogeneity of an image. IDMN weights are the inverse of the Contrast weights (decreasing exponentially from the diagonal $i=j$ in the GLCM). Unlike Homogeneity2, IDMN normalizes the square of the difference between neighboring intensity values by dividing over the square of the total number of discrete intensity values.

18. Inverse Difference (ID)

$$\text{ID} = \sum_{k=0}^{N_g-1} \frac{p_{x-y}(k)}{1+k}$$

ID (a.k.a. Homogeneity 1) is another measure of the local homogeneity of an image. With more uniform gray levels, the denominator will remain low, resulting in a higher overall value.

19. Inverse Difference Normalized (IDN)

$$\text{inverse variance} = \sum_{k=1}^{N_g-1} \frac{p_{x-y}(k)}{k^2}$$

IDN (inverse difference normalized) is another measure of the local homogeneity of an image. Unlike Homogeneity1, IDN normalizes the difference between the neighboring intensity values by dividing over the total number of discrete intensity values.

20. Inverse Variance

$$\text{inverse variance} = \sum_{k=1}^{N_g-1} \frac{p_{x-y}(k)}{k^2}$$

Note that k=0 is skipped, as this would result in a division by 0.

21. Maximum Probability

$$\text{maximum probability}=\max(p(i,j))$$

Maximum Probability is occurrences of the most predominant pair of neighboring intensity values.

22. Sum Average

$$\text{sum average} = \sum_{k=2}^{2N_g} p_{x+y}(k)k$$

Sum Average measures the relationship between occurrences of pairs with lower intensity values and occurrences of pairs with higher intensity values.

23. Sum Entropy

$$\text{sum entropy} = \sum_{k=2}^{2N_g} p_{x+y}(k)\log_2\left(p_{x+y}(k) + \epsilon\right)$$

Sum Entropy is a sum of neighborhood intensity value differences.

24. Sum of Squares

$$\text{sum squares} = \sum_{i=1}^{N_g} \sum_{j=1}^{N_g} (i-\mu_x)^2 p(i,j)$$

Sum of Squares or Variance is a measure in the distribution of neighboring intensity level pairs about the mean intensity level in the GLCM.

Gray Level Size Zone Matrix (GLSZM) Features, N=16

A Gray Level Size Zone (GLSZM) quantifies gray level zones in an image. A gray level zone is defined as the number of connected voxels that share the same gray level intensity. A voxel is considered connected if the distance is 1 according to the infinity norm (26-connected region in a 3D, 8-connected region in 2D). In a gray level size zone matrix $P(i,j)$ the (i,j)th element equals the number of zones with gray level i and size j appear in image. Contrary to GLCM and GLRLM, the GLSZM is rotation independent, with only one matrix calculated for all directions in the ROI.
As a two dimensional example, consider the following 5x5 image, with 5 discrete gray levels:

$$\mathrm{I} = \begin{bmatrix} 5 & 2 & 5 & 4 & 4 \\ 3 & 3 & 3 & 1 & 3 \\ 2 & 1 & 1 & 1 & 3 \\ 4 & 2 & 2 & 2 & 3 \\ 3 & 5 & 3 & 3 & 2 \end{bmatrix}$$

The GLSZM then becomes:

$$P = \begin{bmatrix} 0 & 0 & 0 & 1 & 0 \\ 1 & 0 & 0 & 0 & 1 \\ 1 & 0 & 1 & 0 & 1 \\ 1 & 1 & 0 & 0 & 0 \\ 3 & 0 & 0 & 0 & 0 \end{bmatrix}$$

Let:

- Ng be the number of discreet intensity values in the image
- Ns be the number of discreet zone sizes in the image
- Np be the number of voxels in the image
- Nz be the number of zones in the ROI, which is equal to $\sum_{i=1}^{N_g} \sum_{j=1}^{N_s} P(i,j)$ and $1 \leq Nz \leq Np$
- $\mathbf{P}(i,j)$ be the size zone matrix
- $p(i,j)$ be the normalized size zone matrix, defined as $p(i,j)=\mathbf{P}(i,j)Nz$

1. Small Area Emphasis (SAE)

$$SAE = \frac{\sum_{i=1}^{N_g} \sum_{j=1}^{N_s} \frac{P(i,j)}{j^2}}{N_z}$$

SAE is a measure of the distribution of small size zones, with a greater value indicative of more smaller size zones and more fine textures.

2. Large Area Emphasis (LAE)

$$SAE = \frac{\sum_{i=1}^{N_g} \sum_{j=1}^{N_s} \frac{P(i,j)}{j^2}}{N_z}$$

LAE is a measure of the distribution of large area size zones, with a greater value indicative of more larger size zones and more coarse textures.

3. Gray Level Non-Uniformity (GLN)

$$GLN = \frac{\sum_{i=1}^{N_g} \left(\sum_{j=1}^{N_s} P(i,j)\right)^2}{N_z}$$

GLN measures the variability of gray-level intensity values in the image, with a lower value indicating more homogeneity in intensity values.

4. Gray Level Non-Uniformity Normalized (GLNN)

$$GLNN = \frac{\sum_{i=1}^{N_g} \left(\sum_{j=1}^{N_s} P(i,j)\right)^2}{N_z^2}$$

GLNN measures the variability of gray-level intensity values in the image, with a lower value indicating a greater similarity in intensity values. This is the normalized version of the GLN formula.

5. Size-Zone Non-Uniformity (SZN)

$$SZN = \frac{\sum_{j=1}^{N_s} \left(\sum_{i=1}^{N_g} P(i,j)\right)^2}{N_z}$$

SZN measures the variability of size zone volumes in the image, with a lower value indicating more homogeneity in size zone volumes.

6. Size-Zone Non-Uniformity Normalized (SZNN)

$$SZNN = \frac{\sum_{j=1}^{N_s} \left(\sum_{i=1}^{N_g} P(i,j)\right)^2}{N_z^2}$$

SZNN measures the variability of size zone volumes throughout the image, with a lower value indicating more homogeneity among zone size volumes in the image. This is the normalized version of the SZN formula.

7. Zone Percentage (ZP)

$$\mathrm{ZP} = \frac{N_z}{N_p}$$

ZP measures the coarseness of the texture by taking the ratio of number of zones and number of voxels in the ROI.

8. Gray Level Variance (GLV)

$$\mathrm{GLV} = \sum_{i=1}^{N_g} \sum_{j=1}^{N_s} p(i,j)(i-\mu)^2$$

$$\text{Here, } \mu = \sum_{i=1}^{N_g} \sum_{j=1}^{N_s} p(i,j)i$$

GLV measures the variance in gray level intensities for the zones.

9. Zone Variance (ZV)

$$\mathrm{ZV} = \sum_{i=1}^{N_g} \sum_{j=1}^{N_s} p(i,j)(j-\mu)^2$$

Here, $\mu = \sum_{i=1}^{N_g} \sum_{j=1}^{N_s} p(i,j)i$

ZV measures the variance in zone size volumes for the zones.

10. Zone Entropy (ZE)

$$\mathrm{ZE} = -\sum_{i=1}^{N_g} \sum_{j=1}^{N_s} p(i,j)\log_2\left(p(i,j)+\epsilon\right)$$

Here, ϵ is an arbitrarily small positive number (≈2.2×10−16).

ZE measures the uncertainty/randomness in the distribution of zone sizes and gray levels. A higher value indicates more heterogeneity in the texture patterns.

11. Low Gray Level Zone Emphasis (LGLZE)

$$\mathrm{LGLZE} = \frac{\sum_{i=1}^{N_g} \sum_{j=1}^{N_s} \frac{\mathrm{P}(i,j)}{i^2}}{N_z}$$

LGLZE measures the distribution of lower gray-level size zones, with a higher value indicating a greater proportion of lower gray-level values and size zones in the image.

12. High Gray Level Zone Emphasis (HGLZE)

$$\mathrm{HGLZE} = \frac{\sum_{i=1}^{N_g} \sum_{j=1}^{N_s} \mathrm{P}(i,j)i^2}{N_z}$$

HGLZE measures the distribution of the higher gray-level values, with a higher value indicating a greater proportion of higher gray-level values and size zones in the image.

13. Small Area Low Gray Level Emphasis (SALGLE)

$$\mathrm{SALGLE} = \frac{\sum_{i=1}^{N_g} \sum_{j=1}^{N_s} \frac{\mathrm{P}(i,j)}{i^2 j^2}}{N_z}$$

SALGLE measures the proportion in the image of the joint distribution of smaller size zones with lower gray-level values.

14. Small Area High Gray Level Emphasis (SAHGLE)

$$\text{SAHGLE} = \frac{\sum_{i=1}^{N_g} \sum_{j=1}^{N_s} \frac{\mathrm{P}(i,j)i^2}{j^2}}{N_z}$$

SAHGLE measures the proportion in the image of the joint distribution of smaller size zones with higher gray-level values.

15. Large Area Low Gray Level Emphasis (LALGLE)

$$\text{LALGLE} = \frac{\sum_{i=1}^{N_g} \sum_{j=1}^{N_s} \frac{\mathrm{P}(i,j)j^2}{i^2}}{N_z}$$

LALGLE measures the proportion in the image of the joint distribution of larger size zones with lower gray-level values.

16. Large Area High Gray Level Emphasis (LAHGLE)

$$\text{LAHGLE} = \frac{\sum_{i=1}^{N_g} \sum_{j=1}^{N_s} \mathrm{P}(i,j)i^2j^2}{N_z}$$

LAHGLE measures the proportion in the image of the joint distribution of larger size zones with higher gray-level values.

Gray Level Run Length Matrix (GLRLM) Features, N=16

A Gray Level Run Length Matrix (GLRLM) quantifies gray level runs, which are defined as the length in number of pixels, of consecutive pixels that have the same gray level value. In a gray level run length matrix $\mathbf{P}(i,j|\theta)$, the (i,j)th element describes the number of runs with gray level i and length j occur in the image (ROI) along angle θ.
As a two dimensional example, consider the following 5x5 image, with 5 discrete gray levels:

$$\mathrm{I} = \begin{bmatrix} 5 & 2 & 5 & 4 & 4 \\ 3 & 3 & 3 & 1 & 3 \\ 2 & 1 & 1 & 1 & 3 \\ 4 & 2 & 2 & 2 & 3 \\ 3 & 5 & 3 & 3 & 2 \end{bmatrix}$$

The GLRLM for θ=0, where 0 degrees is the horizontal direction, then becomes:

$$\mathrm{P} = \begin{bmatrix} 1 & 0 & 1 & 0 & 0 \\ 3 & 0 & 1 & 0 & 0 \\ 4 & 1 & 1 & 0 & 0 \\ 1 & 1 & 0 & 0 & 0 \\ 3 & 0 & 0 & 0 & 0 \end{bmatrix}$$

Let:

- Ng be the number of discreet intensity values in the image
- Nr be the number of discreet run lengths in the image
- Np be the number of voxels in the image
- $Nr(\theta)$ be the number of runs in the image along angle θ, which is equal to $\sum_{i=1}^{N_g} \sum_{j=1}^{N_r} \mathrm{P}(i,j|\theta)$ and $1 \le Nr(\theta) \le Np$
- $\mathbf{P}(i,j|\theta)$ be the run length matrix for an arbitrary direction θ
- $p(i,j|\theta)$ be the normalized run length matrix, defined as $p(i,j|\theta)$=$\mathbf{P}(i,j|\theta)Nr(\theta)$

1. Short Run Emphasis (SRE)

$$\text{SRE} = \frac{\sum_{i=1}^{N_g} \sum_{j=1}^{N_r} \frac{\mathrm{P}(i,j|\theta)}{j^2}}{N_r(\theta)}$$

SRE is a measure of the distribution of short run lengths, with a greater value indicative of shorter run lengths and more fine textural textures.

2. Long Run Emphasis (LRE)

$$\text{LRE} = \frac{\sum_{i=1}^{N_g} \quad \sum_{j=1}^{N_r} \text{P}(i,j|\theta)j^2}{N_r(\theta)}$$

LRE is a measure of the distribution of long run lengths, with a greater value indicative of longer run lengths and more coarse structural textures.

3. Gray Level Non-Uniformity (GLN)

$$\text{GLN} = \frac{\sum_{i=1}^{N_g} \quad (\sum_{j=1}^{N_r} \text{P}(i,j|\theta))^2}{N_r(\theta)}$$

GLN measures the similarity of gray-level intensity values in the image, where a lower GLN value correlates with a greater similarity in intensity values.

4. Gray Level Non-Uniformity Normalized (GLNN)

$$\text{GLNN} = \frac{\sum_{i=1}^{N_g} \quad (\sum_{j=1}^{N_r} \text{P}(i,j|\theta))^2}{N_r(\theta)^2}$$

GLNN measures the similarity of gray-level intensity values in the image, where a lower GLNN value correlates with a greater similarity in intensity values. This is the normalized version of the GLN formula.

5. Run Length Non-Uniformity (RLN)

$$\text{RLN} = \frac{\sum_{j=1}^{N_r} \quad (\sum_{i=1}^{N_g} \text{P}(i,j|\theta))^2}{N_r(\theta)}$$

RLN measures the similarity of run lengths throughout the image, with a lower value indicating more homogeneity among run lengths in the image.

6. Run Length Non-Uniformity Normalized (RLNN)

$$\text{RLNN} = \frac{\sum_{j=1}^{N_r} \quad (\sum_{i=1}^{N_g} \text{P}(i,j|\theta))^2}{N_r(\theta)^2}$$

RLNN measures the similarity of run lengths throughout the image, with a lower value indicating more homogeneity among run lengths in the image. This is the normalized version of the RLN formula.

7. Run Percentage (RP)

$$\text{RP} = \frac{N_r(\theta)}{N_p}$$

RP measures the coarseness of the texture by taking the ratio of number of runs and number of voxels in the ROI.

8. Gray Level Variance (GLV)

$$\text{GLV} = \sum_{i=1}^{N_g} \sum_{j=1}^{N_r} p(i,j|\theta)(i-\mu)^2$$

Here, $\mu = \sum_{i=1}^{N_g} \sum_{j=1}^{N_r} p(i,j|\theta)i$ GLV measures the variance in gray level intensity for the runs.

9. Run Variance (RV)

$$\text{RV} = \sum_{i=1}^{N_g} \sum_{j=1}^{N_r} p(i,j|\theta)(j-\mu)^2$$

Here, $\mu = \sum_{i=1}^{N_g} \sum_{j=1}^{N_r} p(i,j|\theta)i$ RV is a measure of the variance in runs for the run lengths.

10. Run Entropy (RE)

$$\mathrm{RE} = -\sum_{i=1}^{N_g}\sum_{j=1}^{N_r} p(i,j|\theta)\log_2\left(p(i,j|\theta)+\epsilon\right)$$

Here, ϵ is an arbitrarily small positive number (≈2.2×10−16).
RE measures the uncertainty/randomness in the distribution of run lengths and gray levels. A higher value indicates more heterogeneity in the texture patterns.

11. Low Gray Level Run Emphasis (LGLRE)

$$\mathrm{LGLRE} = \frac{\sum_{i=1}^{N_g}\sum_{j=1}^{N_r}\frac{\mathrm{P}(i,j|\theta)}{i^2}}{N_r(\theta)}$$

LGLRE measures the distribution of low gray-level values, with a higher value indicating a greater concentration of low gray-level values in the image.

12. High Gray Level Run Emphasis (HGLRE)

$$\mathrm{HGLRE} = \frac{\sum_{i=1}^{N_g}\sum_{j=1}^{N_r}\mathrm{P}(i,j|\theta)i^2}{N_r(\theta)}$$

HGLRE measures the distribution of the higher gray-level values, with a higher value indicating a greater concentration of high gray-level values in the image.

13. Short Run Low Gray Level Emphasis (SRLGLE)

$$\mathrm{SRLGLE} = \frac{\sum_{i=1}^{N_g}\sum_{j=1}^{N_r}\frac{\mathrm{P}(i,j|\theta)}{i^2 j^2}}{N_r(\theta)}$$

SRLGLE measures the joint distribution of shorter run lengths with lower gray-level values.

14. Short Run High Gray Level Emphasis (SRHGLE)

$$SRHGLE = \frac{\sum_{i=1}^{N_g}\sum_{j=1}^{N_r}\frac{\mathrm{P}(i,j|\theta)i^2}{j^2}}{N_r(\theta)}$$

SRHGLE measures the joint distribution of shorter run lengths with higher gray-level values.

15. Long Run Low Gray Level Emphasis (LRLGLE)

$$\mathrm{LRLGLRE} = \frac{\sum_{i=1}^{N_g}\sum_{j=1}^{N_r}\frac{\mathrm{P}(i,j|\theta)j^2}{i^2}}{N_r(\theta)}$$

LRLGLRE measures the joint distribution of long run lengths with lower gray-level values.

16. Long Run High Gray Level Emphasis (LRHGLE)

$$\mathrm{LRHGLRE} = \frac{\sum_{i=1}^{N_g}\sum_{j=1}^{N_r}\mathrm{P}(i,j|\theta)i^2 j^2}{N_r(\theta)}$$

LRHGLRE measures the joint distribution of long run lengths with higher gray-level values.

Neighboring Gray Tone Difference Matrix (NGTDM) Features, N=5

A Neighboring Gray Tone Difference Matrix quantifies the difference between a gray value and the average gray value of its neighbors within distance $\delta\delta$. The sum of absolute differences for gray level ii is stored in the matrix. Let $\mathbf{X}gl$ be a set of segmented voxels and $xgl(jx,jy,jz)\in\mathbf{X}gl$ be the gray level of a voxel at position (jx,jy,jz), then the average gray level of the neighborhood is:

$$\overline{A}_i = \frac{1}{W}\sum_{k_x=-\delta}^{\delta}\sum_{k_y=-\delta}^{\delta}\sum_{k_z=-\delta}^{\delta} x_{gl}(j_x+k_x, j_y+k_y, j_z+k_z),$$

where $(k_x, k_y, k_z) \neq (0,0,0)$ and $x_{gl}(j_x+k_x, j_y+k_y, j_z+k_z) \in X_{gl}$

Here, W is the number of voxels in the neighborhood that are also in $\mathbf{X}gl$.
As a two dimensional example, let the following matrix $\mathbf{I}$ represent a 4x4 image, having 5 discrete grey levels, but no voxels with gray level 4:

$$I = \begin{bmatrix} 1 & 2 & 5 & 2 \\ 3 & 5 & 1 & 3 \\ 1 & 3 & 5 & 5 \\ 3 & 1 & 1 & 1 \end{bmatrix}$$

The following NGTDM can be obtained:

i	n_i	p_i	s_i
1	6	0.375	13.35
2	2	0.125	2.00
3	4	0.25	2.63
4	0	0.00	0.00
5	4	0.25	10.075

6 pixels have gray level 1, therefore:

$$s_1 = |1-10/3| + |1-30/8| + |1-15/5| + |1-13/5| + |1-15/5| + |1-11/3| = 13.35$$

For gray level 2, there are 2 pixels, therefore:

$$s_2 = |2-15/5| + |2-15/5| = 2$$

Similar for gray values 3 and 5:

$$s_3 = |3-12/5| + |3-18/5| + |3-20/8| + |3-5/3| = 3.03 s_5$$
$$= |5-14/5| + |5-18/5| + |5-20/8| + |5-11/5| = 10.075$$

Let:
ni be the number of voxels in Xgl with gray level i
Nv,p be the total number of voxels in Xgl and equal to $\sum ni$ (i.e. the number of voxels with a valid region; at least 1 neighbor). $Nv,p\leq Np$, where Np is the total number of voxels in the ROI.
pipi be the gray level probability and equal to ni/Nv

$$s_i = \begin{cases} \sum^{n_i} |i-\overline{A}_i| & \text{for} \quad n_i \neq 0 \\ 0 & \text{for} \quad n_i = 0 \end{cases}$$

s be the sum of absolute differences for gray level i
Ng be the number of discreet gray levels
Ng,p be the number of gray levels where pi≠0

1. Coarseness

$$Coarseness = \frac{1}{\sum_{i=1}^{N_g} p_i s_i}$$

Coarseness is a measure of average difference between the center voxel and its neighborhood and is an indication of the spatial rate of change. A higher value indicates a lower spatial change rate and a locally more uniform texture.

2. Contrast

$$Contrast = \left(\frac{1}{N_{g,p}(N_{g,p}-1)}\sum_{i=1}^{N_g}\sum_{j=1}^{N_g} p_i p_j (i-j)^2\right)\left(\frac{1}{N_{v,p}}\sum_{i=1}^{N_g} s_i\right), \text{where } p_i \neq 0, p_j \neq 0$$

Contrast is a measure of the spatial intensity change, but is also dependent on the overall gray level dynamic range. Contrast is high when both the dynamic range and the spatial change rate are high, i.e. an image with a large range of gray levels, with large changes between voxels and their neighborhood.

3. Busyness

$$Busyness = \frac{\sum_{i=1}^{N_g} p_i s_i}{\sum_{i=1}^{N_g}\sum_{j=1}^{N_g} |ip_i - jp_j|}, \text{where } p_i \neq 0, p_j \neq 0$$

A measure of the change from a pixel to its neighbor. A high value for busyness indicates a 'busy' image, with rapid changes of intensity between pixels and its neighborhood.

4. Complexity

$$Complexity = \frac{1}{N_{v,p}}\sum_{i=1}^{N_g}\sum_{j=1}^{N_g} |i-j| \frac{p_i s_i + p_j s_j}{p_i + p_j}, \text{where } p_i \neq 0, p_j \neq 0$$

An image is considered complex when there are many primitive components in the image, i.e. the image is non-uniform and there are many rapid changes in gray level intensity.

5. Strength

$$Strength = \frac{\sum_{i=1}^{N_g}\sum_{j=1}^{N_g} (p_i + p_j)(i-j)^2}{\sum_{i=1}^{N_g} s_i}, \text{where } p_i \neq 0, p_j \neq 0$$

Strength is a measure of the primitives in an image. Its value is high when the primitives are easily defined and visible, i.e. an image with slow change in intensity but more large coarse differences in gray level intensities.

Gray Level Dependence Matrix (GLDM) Features, N=14

A Gray Level Dependence Matrix (GLDM) quantifies gray level dependencies in an image. A gray level dependency is defined as the number of connected voxels within distance δδ that are dependent on the center voxel. A neighboring voxel with gray level j is considered dependent on center voxel with gray level i if $|i-j| \le \alpha$. In a gray level dependence matrix $\mathbf{P}(i,j)$ the $(i,j)^{th}$ element describes the number of times a voxel with gray level i with j dependent voxels in its neighborhood appears in image.

As a two dimensional example, consider the following 5x5 image, with 5 discrete gray levels:

$$I = \begin{bmatrix} 5 & 2 & 5 & 4 & 4 \\ 3 & 3 & 3 & 1 & 3 \\ 2 & 1 & 1 & 1 & 3 \\ 4 & 2 & 2 & 2 & 3 \\ 3 & 5 & 3 & 3 & 2 \end{bmatrix}$$

For α=0 and δ=1, the GLDM then becomes:

$$P = \begin{bmatrix} 0 & 1 & 2 & 1 \\ 1 & 2 & 3 & 0 \\ 1 & 4 & 4 & 0 \\ 1 & 2 & 0 & 0 \\ 3 & 0 & 0 & 0 \end{bmatrix}$$

Let:

- Ng be the number of discreet intensity values in the image
- Nd be the number of discreet dependency sizes in the image
- Nz be the number of dependency zones in the image, which is equal to $\sum Ngi{=}1\sum Ndj{=}1\mathbf{P}(i,j)$
- $\mathbf{P}(i,j)$ be the dependence matrix
- $p(i,j)$ be the normalized dependence matrix, defined as $p(i,j){=}\mathbf{P}(i,j)Nz$

1. Small Dependence Emphasis (SDE)

$$SDE = \frac{\sum_{i=1}^{N_g} \sum_{j=1}^{N_d} \frac{\mathrm{P}(i,j)}{i^2}}{N_z}$$

A measure of the distribution of small dependencies, with a greater value indicative of smaller dependence and less homogeneous textures.

2. Large Dependence Emphasis (LDE)

$$LDE = \frac{\sum_{i=1}^{N_g} \sum_{j=1}^{N_d} \mathrm{P}(i,j) j^2}{N_z}$$

A measure of the distribution of large dependencies, with a greater value indicative of larger dependence and more homogeneous textures.

3. Gray Level Non-Uniformity (GLN)

$$GLN = \frac{\sum_{i=1}^{N_g} \left(\sum_{j=1}^{N_d} \mathrm{P}(i,j)\right)^2}{N_z}$$

Measures the similarity of gray-level intensity values in the image, where a lower GLN value correlates with a greater similarity in intensity values.

4. Dependence Non-Uniformity (DN)

$$DN = \frac{\sum_{j=1}^{N_d} \left(\sum_{i=1}^{N_g} \mathrm{P}(i,j)\right)^2}{N_z}$$

Measures the similarity of dependence throughout the image, with a lower value indicating more homogeneity among dependencies in the image.

5. Dependence Non-Uniformity Normalized (DNN)

$$DNN = \frac{\sum_{j=1}^{N_d} \left(\sum_{i=1}^{N_g} \mathrm{P}(i,j)\right)^2}{N_z^2}$$

Measures the similarity of dependence throughout the image, with a lower value indicating more homogeneity among dependencies in the image. This is the normalized version of the DLN formula.

6. Gray Level Variance (GLV)

$$GLV = \sum_{i=1}^{N_g} \sum_{j=1}^{N_d} p(i,j)(i-\mu)^2 \text{, where} \mu = \sum_{i=1}^{N_g} \sum_{j=1}^{N_d} ip(i,j)$$

Measures the variance in grey level in the image.

7. Dependence Variance (DV)

$$DV = \sum_{i=1}^{N_g} \sum_{j=1}^{N_d} p(i,j)(j-\mu)^2 \text{, where } \mu = \sum_{i=1}^{N_g} \sum_{j=1}^{N_d} jp(i,j)$$

Measures the variance in dependence size in the image.

8. Dependence Entropy (DE)

$$DependenceEntropy = -\sum_{i=1}^{N_g} \sum_{j=1}^{N_d} p(i,j)\log_2 (p(i,j)+\epsilon)$$

9. Low Gray Level Emphasis (LGLE)

$$LGLE = \frac{\sum_{i=1}^{N_g} \sum_{j=1}^{N_d} \frac{\mathrm{P}(i,j)}{i^2}}{N_z}$$

Measures the distribution of low gray-level values, with a higher value indicating a greater concentration of low gray-level values in the image.

10. High Gray Level Emphasis (HGLE)

$$HGLE = \frac{\sum_{i=1}^{N_g} \sum_{j=1}^{N_d} \mathrm{P}(i,j)i^2}{N_z}$$

Measures the distribution of the higher gray-level values, with a higher value indicating a greater concentration of high gray-level values in the image.

11. Small Dependence Low Gray Level Emphasis (SDLGLE)

$$SDLGLE = \frac{\sum_{i=1}^{N_g} \sum_{j=1}^{N_d} \frac{\mathrm{P}(i,j)}{i^2 j^2}}{N_z}$$

Measures the joint distribution of small dependence with lower gray-level values.

12. Small Dependence High Gray Level Emphasis (SDHGLE)

Measures the joint distribution of small dependence with higher gray-level values.

13. Large Dependence Low Gray Level Emphasis (LDLGLE)

$$LDLGLE = \frac{\sum_{i=1}^{N_g} \sum_{j=1}^{N_d} \frac{\mathrm{P}(i,j)j^2}{i^2}}{N_z}$$

Measures the joint distribution of large dependence with lower gray-level values.

14. Large Dependence High Gray Level Emphasis (LDHGLE)

$$LDHGLE = \frac{\sum_{i=1}^{N_g} \sum_{j=1}^{N_d} \mathrm{P}(i,j)i^2 j^2}{N_z}$$

Measures the joint distribution of large dependence with higher gray-level values.
