# Supplementary material for: A Multiparametric Method Based on Clinical and CT-Based Radiomics to Predict the Expression of p53 and VEGF in Patients With Spinal Giant Cell Tumor of Bone
Source: Front Oncol. 2022 Jun 21;12:894696. doi: 10.3389/fonc.2022.894696 (PMC9253421; doi:10.3389/fonc.2022.894696)
Supplement: Supplementary file 3 [file DataSheet_3.pdf]

Supplementary Material

Supplementary Part 3

Abbreviations:

AUC: Area Under ROC Curve

CT: Computed Tomography

DCE: Dynamic Contrast-Enhanced

DWI: Diffusion Weighted Imaging

GCT: Giant Cell Tumor

GCTB: Giant Cell Tumor of Bone

GLCM: Gray-Level Co-Occurrence Matrix

GLDM: Gray Level Dependence Matrix

GLRLM: Gray Level Run Length Matrix

IDH1: Isocitrate DeHydrogenase 1

IHC: Immunohistochemistry

MRI: Magnetic Resonance Imaging

PFS: Progression-Free Survival

ROC: Receiver Operating Characteristic

ROI: Region of Interest

SINS: Spinal Instability Neoplastic Score

SVM: Support Vector Machine

TES: Total En bloc Spondylectomy

VAS: Visual Analog Scale

VEGF: Vascular Endothelial Growth Factor

WHO: World Health Organization
