# Supplementary material for: A Multiparametric Method Based on Clinical and CT-Based Radiomics to Predict the Expression of p53 and VEGF in Patients With Spinal Giant Cell Tumor of Bone
Source: Front Oncol. 2022 Jun 21;12:894696. doi: 10.3389/fonc.2022.894696 (PMC9253421; doi:10.3389/fonc.2022.894696)
Supplement: Supplementary file 4 [file DataSheet_4.pdf]

Supplementary Material

Supplementary Part 4

This part mainly supplements the survival analysis of important factors. Independent prognostic factors were identified using univariate and multivariate analysis. Univariate analysis was carried out using log rank test and multivariate analysis were adjusted by Cox regression analysis, and a forest plot were conducted for PFS. The analysis process was performed using R 3.6.3 software (The R Foundation for Statistical Computing, Vienna, Austria). $P<0.05$ was accepted as statistically significant.

Supplementary Figure 3

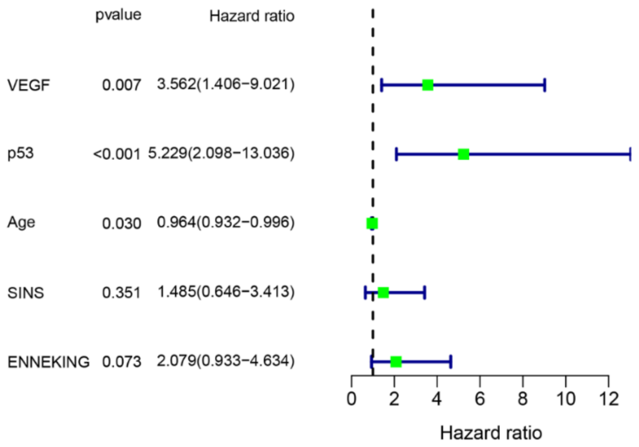

Supplementary Figure 3. Cox univariate regression analysis was used to 5 factors, including VEGF, p53, age, SINS and Enneking stage. P-value, hazard radio (HR) and 95% confidence interval (CI) are listed in a forest plot. p-value < 0.05 for age, p53 and VEGF.

Supplementary Figure 4

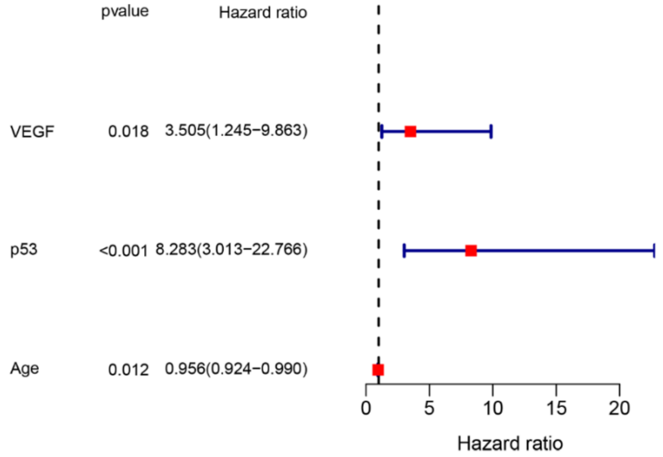

Supplementary Figure 4. Cox multivariate regression analysis and forest plot of hazard ratios with 95% CI for 3 factors, including VEGF, p53 and age (p < 0.05). Among them, the HR of age is less than 1, which may be a relatively weak protective factor, while p53 and VEGF are risk factors.

Supplementary Figure 5

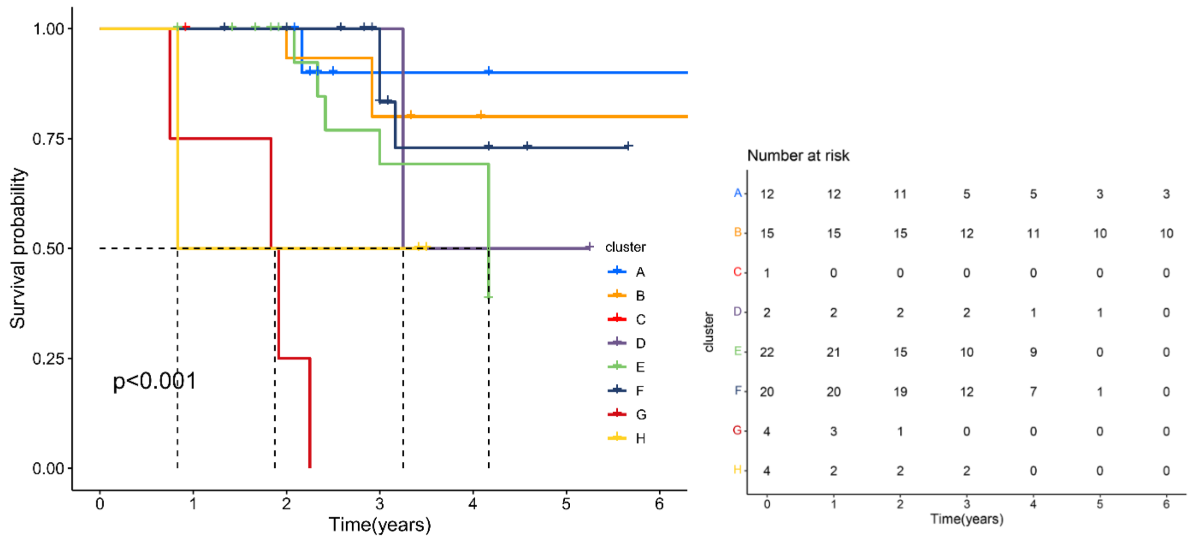

Supplementary Figure 5. Multivariate Cox regression analysis for PFS according to baseline characteristics. During the entire follow-up process, more than 50% of patients progressed in groups D, E, G, and H. The vertical dotted line in the picture indicates the median survival of these four groups, which were 3.250, 4.167, 1.875 and 0.833 years respectively. In order to ensure the visualization of the curve, the age variable is grouped, and 30 years old are used for binary classification. The details of clusters are showed in Table 4.

Supplementary Table 4. The details of clusters in multivariate Cox regression analysis.

Cluster	*VEGF*	*p53*	*Age*
A	0	0	≤30
B	0	0	>30
C	0	1	≤30
D	0	1	>30
E	1	0	≤30
F	1	0	>30
G	1	1	≤30
H	1	1	>30

Note: 0 for low-VEGF/wild-type p53 groups, 1 for high-VEGF/mutant p53 groups.
